# Multi-focal ultrasound neuromodulation to the dorsal anterior cingulate cortex disrupts behavioural and neural pain processing

**DOI:** 10.1038/s41467-026-72934-3

**Published:** 2026-05-09

**Authors:** Sophie Clarke, Samuel Mugglestone, Mathilde Lojkiewiez, Joshua Marquez, Nadège Bault, Elsa Fouragnan, Sam Hughes

**Affiliations:** 1 School of Psychology, Faculty of Health, University of Plymouth, Plymouth, UK; 2 Brain Research and Imaging Centre, Faculty of Health, University of Plymouth, Plymouth, UK; 3 Department of Clinical and Biomedical Sciences, Faculty of Health and Life Sciences, University of Exeter, Exeter, UK

**Keywords:** Neural circuits, Preclinical research

## Abstract

Transcranial ultrasound stimulation (TUS) is a promising non-invasive neuromodulation technique for pain-related deep brain regions. This study aimed to investigate neural mechanisms underlying TUS effects on pain processing using neuroimaging. Thirty-two healthy participants underwent two double-blind, randomised sessions (active or sham). A tonic cold stimulus was applied during multifocal TUS applied to the dorsal anterior cingulate cortex (dACC), and during functional magnetic resonance imaging (fMRI) and magnetic resonance spectroscopy (MRS). While no significant main effect on pain intensity was observed, active TUS showed a significantly greater reduction in pain ratings between 28- and 55-minutes post-stimulation, suggesting a delayed analgesic effect. Active TUS altered sensory encoding, disrupting the relationship between temperature and pain intensity. There was increased functional connectivity between the dACC and the supplementary motor area, pre-motor cortex, mid-ACC and supramarginal gyrus, and altered salience network connectivity. Overall, these findings suggest dACC-TUS has multidimensional effects across behavioural and neural aspects of pain processing, supporting its potential therapeutic value.

## Abstract (web summary)

This study shows that transcranial ultrasound stimulation (TUS) changes how the brain processes pain, altering sensory encoding and connectivity in key networks. These findings provide insight into the neural mechanisms underlying both pain perception and TUS effects.

## Introduction

Transcranial ultrasound stimulation (TUS) enables precise, targeted neuromodulation of both cortical and deep brain regions, and it has been demonstrated across multiple studies that TUS can elicit behavioural changes and alter neuronal activity in humans, in both preclinical and clinical studies^1–4^. TUS is a particularly promising approach for pain neuromodulation given the involvement of deep cortical and sub-cortical regions in the pain network, such as the dorsal anterior cingulate cortex (dACC), insular cortex, thalamus or amygdala^5^. TUS protocols can be classified as either online, causing short-term effects during or immediately after stimulation, or offline, producing longer-lasting effects persisting beyond the stimulation period^1^. Previous studies have demonstrated the effectiveness of both online and offline TUS in modulating pain, with online TUS applied to the dACC shown to reduce pain ratings to acute heat stimuli as well as alter autonomic responses and the amplitude of contact heat-evoked potentials, and offline TUS applied to the anterior thalamus shown to reduce thermal pain thresholds, both in healthy humans^6,7^. An early study in chronic pain patients showed offline TUS applied to the posterior frontal cortex resulted in significant improvements in pain ratings and mood 40 min after stimulation^8^. The delayed effects of offline TUS are particularly relevant in pain research, providing potential for translating these protocols into clinical interventions aimed at achieving long-lasting therapeutic benefits.

The dACC is a particularly relevant brain region for pain neuromodulation. It has a central role in pain processing, is involved in both ascending and descending pathways and plays a role in emotional and cognitive processing^9^. Importantly, targeting the dACC using deep brain stimulation (DBS) has been shown to be effective in treating chronic pain conditions, with improvements in patient-reported pain scores and quality of life^9–11^. In a cohort of chronic pain patients, TUS applied to the dACC has been shown to significantly reduce pain immediately after stimulation, with improvement sustained for up to 7 days post-treatment^12^.

The underlying neural basis for these improvements in reported pain following TUS to the dACC is not fully understood. This study aimed to further our understanding of the neural basis for altered pain processing following TUS to the dACC by using functional magnetic resonance imaging (fMRI) and magnetic resonance spectroscopy (MRS) techniques to explore functional connectivity changes and neurochemical changes, respectively. Gaining this mechanistic understanding of the neural basis of effects is valuable in progressing the utility of TUS for pain; by identifying the brain regions and circuits involved (supporting optimal target selection) and by understanding whether TUS-induced effects can produce longer-term analgesia through network reorganisation. Crucially, this enhanced understanding of the neural basis of effects also enables a deeper insight into how TUS could be optimally adapted to treat different pain conditions, given the significant heterogeneity in the neural circuits and mechanisms involved in different types of pain. TUS has been shown in several fMRI studies to illicit changes in functional connectivity with the brain region targeted, as well as larger-scale changes in connectivity across brain networks^4,13–16^. MRS has also been previously employed to investigate neurochemical changes resulting from TUS, showing that TUS can result in altered GABA and glutamate (Glx) concentrations at the application site for some brain regions^4,17,18^, although one study also reported that GABA concentration was not altered following TUS to the dACC^4^. Multiple factors, such as the brain region, the current activation state or tissue composition, may influence TUS effects on neurochemical concentrations and further research is needed in this area.

For the current study, we investigated TUS-induced changes in participant-reported pain ratings, functional connectivity and neurochemistry during exposure of the right hand to a tonic cold pain stimulus. TUS was applied to multiple sites of the dACC during tonic pain to specifically elicit neuronal changes related to an active pain state in that brain region. TUS-induced effects were investigated at two time points during tonic cold pain, at 28 min post-TUS using fMRI measures of functional connectivity, and 55 min post-TUS using MRS measures of neurochemistry. We hypothesised that TUS would result in altered pain perception, accompanied by underlying changes in functional connectivity between brain regions known to be involved in pain processing, as well as changes in neurochemical concentrations within the dACC. Specifically, since TUS has been shown to result in analgesia in previous studies, we hypothesised that the behavioural measure of pain perception would show decreased pain ratings. During painful heat stimulation, it has been shown that ACC Glx increases and GABA decreases^19^, therefore, during TUS-induced analgesia (decreased pain intensity), we hypothesised that ACC Glx would decrease and GABA would increase.

## Results

**Fig. 1:**
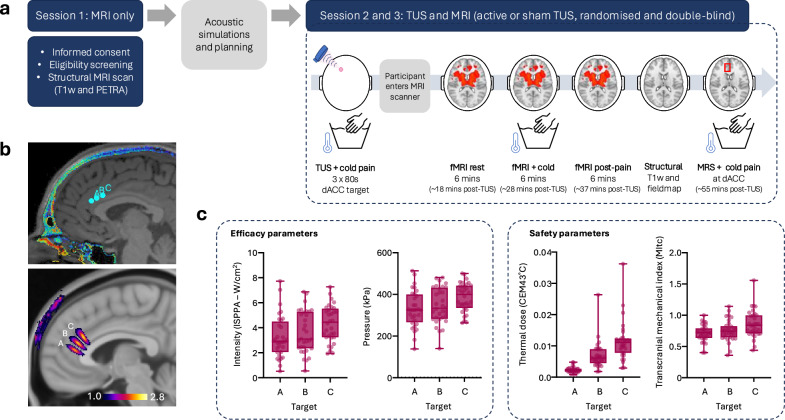
Study design and TUS post-stimulation simulation results (*N* = 32 participants). **a** Participants completed three sessions; in the first session, T1-weighted and PETRA scans were acquired for neuronavigation and acoustic planning, then in sessions 2 and 3, they received either active TUS to the dACC or sham, followed by completing fMRI and MRS scans. **b** This multi-focal TUS intervention involved application of TUS to 3 target sites within the dACC, which are shown in the top panel labelled A, B and C. Average stimulation intensity (I_SPPA_) simulated using k-Plan software (BrainBox, Inc.) across all participants is shown in the bottom panel. **c** Efficacy and safety parameters from post-stimulation simulations shown for all participants for intensity (I_SPPA_) and pressure (kPa) in the dACC, thermal dose (CEM43 °C) in soft tissues, and transcranial mechanical index (MItc) across the three targets. Box plots are defined such that the box represents the interquartile range (25th–75th percentiles), the central line denotes the median, and the whiskers extend to the minimum and maximum values. Source data are provided as a Source Data file.

In total, 35 healthy participants were recruited to take part in the study, which investigated the effects of multi-focal active TUS vs. sham TUS on the dACC on pain responses to a tonic cold stimulus (see study design summarised in Fig. 1a). Two participants were excluded after the first visit due to previously experiencing severe concussion (*n* = 1), a contraindication for TUS, and due to an incidental finding in the first MRI (*n* = 1). Therefore, 33 participants completed the study (mean age 26.3 ± 10.2 years, range 21–66, 19 female, 14 male, sex and gender aligned by self-report). One dataset was excluded due to technical issues with the ultrasound, resulting in *n* = 32 participants for the behavioural dataset, and three further datasets were excluded due to an artefact following scanner software upgrade, resulting in *n* = 29 participants for the fMRI dataset (mean age 25.6 ± 9.5 years, range 21–66, 19 female, 10 male).

### TUS post-stimulation simulations for assessment of efficacy and safety

Targeted areas and average stimulation intensity (*I*_SPPA_) across all participants are shown in Fig. 1b. Detailed acoustic simulation parameters and outputs for all study participants can be found in Supplementary Materials [Table 1] and are summarised in Fig. 1c. Overall, there was good target engagement, with a mean ISPPA in the dACC of 3.7 ± 1.7w/cm^2^ across all participants. Importantly, transcranial Mechanical Index (MItc) remained below 1.9, and the Cumulative Equivalent Minutes at 43 °C (CEM43), a metric reflecting both duration and intensity of heating relative to 43 °C (the critical threshold for thermal cell damage), remained well below the safety threshold of 0.25^20^.

### Behavioural results

**Fig. 2:**
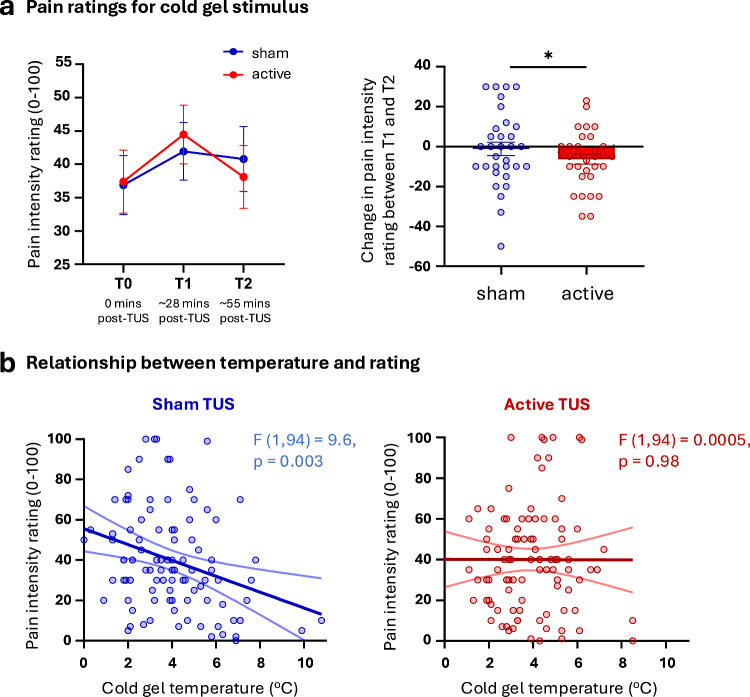
Pain intensity ratings to tonic cold stimulus (*N* = 32 participants). **a** Average pain intensity ratings for each of the three time points (left) and plot showing significant difference in the change in pain rating between T1 and T2 for active TUS compared to sham TUS (right; robust linear mixed effects model, Δ = –8.15, z(inf) = −2.02, *p* = 0.043, *d* = 0.79, 95% CI [−16.05, −0.26]). Fixed effects were tested using two-tailed tests. Post-hoc pairwise contrasts were conducted using estimated marginal means; *p*-values were adjusted for multiple comparisons using Tukey’s method. Data are presented as mean values, and error bars show the SEM. Blue = sham TUS, red = active TUS. * represents *p* < 0.05. Source data are provided as a Source Data file. **b** Relationship between gel temperature and pain intensity ratings pooled for all three time points and plotted for both sham TUS (left) and active TUS (right), showing the expected relationship between temperature and pain intensity is present in the sham condition (simple linear regression; *F*(1, 94) = 9.60, *p* = 0.003, *β* = −3.93, 95% CI [ − 6.45, − 1.41] but no longer present in the active TUS session (simple linear regression; *F*(1, 94) = 0.0005, *p* = 0.980, *β* = −0.035, 95% CI [−3.301, 3.231]). Plots show the regression line (bold line) and the 95% confidence intervals (paler line). Blue = sham TUS, red = active TUS. Source data are provided as a Source Data file.

Pain intensity ratings were given after approximately 6 min tonic cold stimulus at three time points: T0—during active or sham TUS to the dACC; T1—during the resting state fMRI scan; and T2—during the MRS scan. Linear mixed-effects modelling showed no significant main differences between active and sham TUS at any individual time point (T0; *t*(148) = −0.03, *p* = 0.976, *d* = −0.008, 95% CI [−5.74, 5.57]), T1; *t*(148) = −0.80, *p* = 0.425, *d* = −0.21, 95% CI [−8.31, 3.52]) or T2; *t*(148) = 1.81, *p* = 0.072, *d* = 0.49, 95% CI [−0.50, 11.70]). Further exploratory analysis using a robust linear mixed-effects model showed the reduction in pain ratings from T1 to T2 was significantly greater in the active group compared to sham (Δ = –8.15, z(inf) = −2.02, *p* = 0.043, *d* = 0.79, 95% CI [−16.05, −0.26]), potentially suggesting a delayed analgesic effect of TUS. This is shown in Fig. 2a.

A feature of the cold gelled-water stimulus used in this study was that there was variation in the temperature of the stimulus overall. This enabled the investigation of temperature sensitivity across the pooled dataset. Simple linear regression was used to evaluate the relationship between temperature and pain intensity rating across all participants and all time points, for both the active TUS and sham TUS sessions. For sham TUS, there was a statistically significant relationship between temperature and pain intensity rating (*F*(1, 94) = 9.60, *p* = 0.003, *β* = −3.93, 95% CI [ − 6.45, −1.41]) whereas for active TUS sensitivity or intensity encoding to cold stimulus may have been altered as this expected relationship was no longer statistically significant (*F*(1, 94) = 0.0005, *p* = 0.980, *β* = −0.035, 95% CI [−3.301, 3.231]). These results are shown in Fig. 2b. Robust linear regression confirmed the patterns observed. In the sham condition, temperature was negatively associated with rating (*t*(94) = − 3.15, *p* = 0.002, *β* = −4.04, 95% CI [ − 6.55, −1.53]), consistent with the original result. In the active condition, there was no association (*t*(94) = − 0.29, *p* = 0.780, *β* = −0.29, 95% CI [ − 2.25, 1.67]), and in both cases a few moderately extreme points were down-weighted without altering the overall conclusion, confirming that the observed effects (or lack thereof) were not driven by outliers. Control analyses were completed to ensure there were no significant differences in temperature between the active and sham conditions that may influence the results. This confirmed that there were no significant differences between the temperature of the gel stimulus between the active and sham conditions for either all three time points pooled data (paired *t*-test, *t*(df) = −0.54, *p* = 0.59, *R*² = 0.003, 95% CI [ − 0.55, 0.31]), or for any of the time points individually (T0 paired *t*-test; *t*(df) = 0.31, *p* = 0.76, *R*² = 0.0031, 95% CI [ − 0.87, 0.64], T1 paired *t*-test; *t*(df) = 0.30, *p* = 0.76, *R*² = 0.0029, 95% CI [ − 1.04, 0.77], T2 paired *t*-test; *t*(df) = 0.32, *p* = 0.75, *R*² = 0.0032, 95% CI [ − 0.74, 0.54]). Plots are shown in Supplementary Fig. 2.

### Imaging results: seed-based functional connectivity and salience network connectivity changes during tonic cold pain

**Fig. 3:**
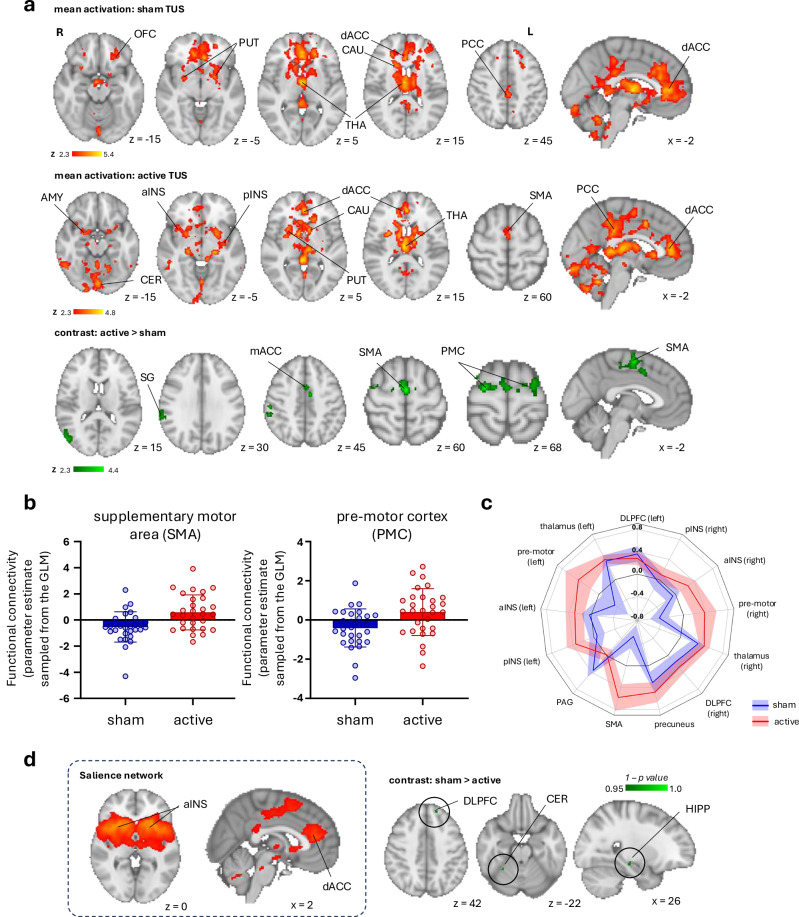
Seed-based functional connectivity and salience network connectivity results (*N* = 29 participants). **a** Whole-brain seed-based functional connectivity maps with the dACC seed in the sham TUS condition (top row), the active TUS condition (middle row), and for the contrast active TUS condition >sham condition (bottom row). Maps were generated using a voxelwise general linear model implemented in FSL FEAT with group-level analyses conducted using mixed-effects modelling (FLAME). Statistical inference was based on voxelwise *t*-tests, and all tests were two-sided. Resulting statistical maps were thresholded using a cluster-forming threshold of *Z* > 2.3, with cluster-level correction for multiple comparisons at *p* < 0.05 (family-wise error corrected). **b** Illustrative plots showing the altered connectivity with dACC seed region for the supplementary motor area (left), the pre-motor cortex (right). Plots show the functional connectivity (parameter estimate from the GLM) sampled from each region, for the sham TUS (blue) and active TUS (red) conditions. Source data are provided as a Source Data file. **c** Illustrative radar plot showing dACC functional connectivity (parameter estimate from the GLM) sampled from brain regions involved in pain processing, including the two regions that showed significantly altered connectivity in the whole-brain analysis, plus the bilateral dorsolateral prefrontal cortex, thalamus, anterior/posterior insular cortices and the periaqueductal grey and precuneus. Blue = sham TUS, red = active TUS. Source data are provided as a Source Data file. **d** Salience network was identified using independent component analysis (ICA) carried out on the full dataset (shown in dotted inset), and subsequent group analysis comparing subject-specific network maps for active TUS and sham conditions showed altered salience network connectivity with the left DLPFC, right cerebellum and right hippocampus. MNI co-ordinates are shown for each image slice, and error bars show the SEM. OFC orbitofrontal cortex, PUT putamen, CAU caudate, dACC dorsal anterior cingulate cortex, PCC posterior cingulate cortex, AMY amygdala, CER cerebellum, pINS posterior insular cortex, aINS anterior insular cortex, SMA supplementary motor cortex, SG supramarginal gyrus, mACC mid-anterior cingulate cortex, PMC pre-motor cortex, DLPFC dorsolateral prefrontal cortex, PAG periaqueductal grey.

First, a seed-based connectivity approach was utilised to assess functional connectivity with the dACC target region during tonic cold pain. Whole-brain group average maps for mean activation during both the sham and active TUS conditions are shown in Fig. 3a, showing the connectivity profile of the dACC during the tonic cold stimulus, which involves the thalamus, posterior cingulate cortex (PCC), caudate and putamen in both conditions. Differentially, there is connectivity between the dACC and the orbitofrontal cortex (OFC) in the sham condition and with the anterior and posterior insular cortex, amygdala and supplementary motor area (SMA) in the active TUS condition. A whole-brain, mixed-effects analysis (*Z* > 2.3, *p* < 0.05) was conducted to compare active and sham TUS conditions, which showed there was increased connectivity between the dACC and pain-related brain regions, including the SMA, pre-motor cortex (PMC), mid-ACC and the supramarginal gyrus (SG), following active TUS compared to sham. This is shown in the bottom panel of Fig. 3a, with individual participant data plotted in Fig. 3b to illustrate this result, showing that this reflects a change from negative dACC-SMA and dACC-PMC connectivity in the sham condition to positive connectivity in the active TUS condition. In addition to these two regions, functional connectivity coefficients for further regions of interest, the bilateral thalamus, anterior/posterior insular cortices, and dorsolateral prefrontal cortex (DLPFC), and the periaqueductal grey (PAG) and precuneus, were also plotted (Fig. 3c). This illustrates the overall shift in the connectivity profile of the dACC, with the increased dACC-SMA and dACC-PMC connectivity accompanied by increased connectivity with the anterior insular and decreased connectivity with the PAG in the active TUS condition compared to sham, while dACC-thalamus and dACC-DLPFC connectivity was unchanged between conditions. These regions were selected as they are involved in pain responses and known to be connected to the dACC, with a focus on the left side as the pain stimulus was applied to the right hand.

Next, independent component analysis (ICA) and dual regression approaches were used to investigate changes in salience network connectivity—a well-defined brain network of which the dACC is a core node. Subject-specific salience network maps were compared for the active TUS and sham TUS conditions, and although there were no connectivity changes within the network itself, there was significantly decreased connectivity between the salience network and the left DLPFC, cerebellum and right hippocampus following active-TUS compared to sham-TUS. This is shown in Fig. 3d.

### Control analyses to assess the specificity of TUS effects to the dACC region only

**Fig. 4:**
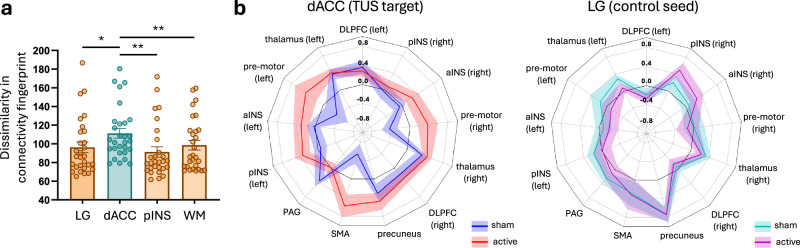
Specificity of TUS effects to dACC connectivity only (*N* = 29 participants). **a** Plot showing dissimilarity in connectivity fingerprints between the active and sham conditions for four regions: the dACC (TUS target)—shown in pale blue and three control regions; the LG, pINS and WM—shown in orange. Paired two-sided t-tests were performed comparing the dACC to each control region: LG; *p* = 0.0123, *t*(28) = –2.676, pINS; *p *= 0.0004, *t*(27) = –4.062, and WM; *p* = 0.0023, t(28) = –3.362, with no adjustments for multiple comparisons. *p < 0.05, **p < 0.01. Source data are provided as a Source Data file. **b** Illustrative radar plots showing dACC (TUS target) and LG (control seed) connectivity during active and sham TUS conditions, with brain regions involved in pain processing, including the two regions that showed significantly altered connectivity in the dACC whole-brain analysis, plus the bilateral dorsolateral prefrontal cortex, thalamus, anterior/posterior insular cortices and the periaqueductal grey and precuneus. For the dACC seed, blue = sham TUS, red = active TUS, and for the LG seed, cyan = sham TUS and pink = active TUS. Source data are provided as a Source Data file. Error bars show the SEM. LG lingual gyrus, dACC dorsal anterior cingulate cortex, pINS posterior insular cortex, WM white matter, DLPFC dorsolateral prefrontal cortex, aINS anterior insular cortex, SMA supplementary motor cortex, PAG periaqueductal grey.

To investigate the specificity and causal link between TUS and the reported neural effects, specifically the observed changes in dACC functional connectivity during tonic cold pain, further exploratory analyses were conducted to assess whether the observed modulation of the dACC connectivity fingerprint was specific to the stimulated region and not driven by general session effects. Computed connectivity fingerprints for the dACC (region of interest) and three control regions (regions showing the weakest functional connectivity with the dACC under the sham condition; the lingual gyrus (LG), white matter (WM) and the posterior insular cortex (pINS)) showed that the dissimilarity in connectivity fingerprints between the active and sham conditions was significantly greater in the dACC compared with other regions that were not directly sonicated (LG, WM, pINS; Fig. 4a). Specifically, pairwise comparisons showed higher session-related dissimilarity in the dACC relative to all control regions (LG: *t*(28) = −2.68, *p* = 0.01, *d* = −0.50, 95% CI [−26.25, −3.49]; WM: *t*(28) = −3.36, *p* = 0.002, *d* = −0.62, 95% CI [−20.27, −4.92]; pINS: *t*(27) = −4.06, *p* = 0.0004, *d* = −0.77, 95% CI [−26.92, −8.85]). These results indicate that the observed modulation of the connectivity fingerprint is localised to the dACC, the site of sonication, and is not present in regions outside the TUS focus. In addition, we conducted a further whole-brain seed-based analysis using an alternate (control) seed region. We selected LG as the seed region, since it is not anatomically close to the dACC or strongly functionally connected. There were no significant differences in connectivity at the whole-brain level, and as shown in Fig. 4b, the functional connectivity between the LG seed region and the same ROIs included in previous analyses was not altered between the active and sham TUS conditions, adding further evidence for TUS specificity in observed dACC connectivity changes.

### State-dependency of TUS effects

**Fig. 5:**
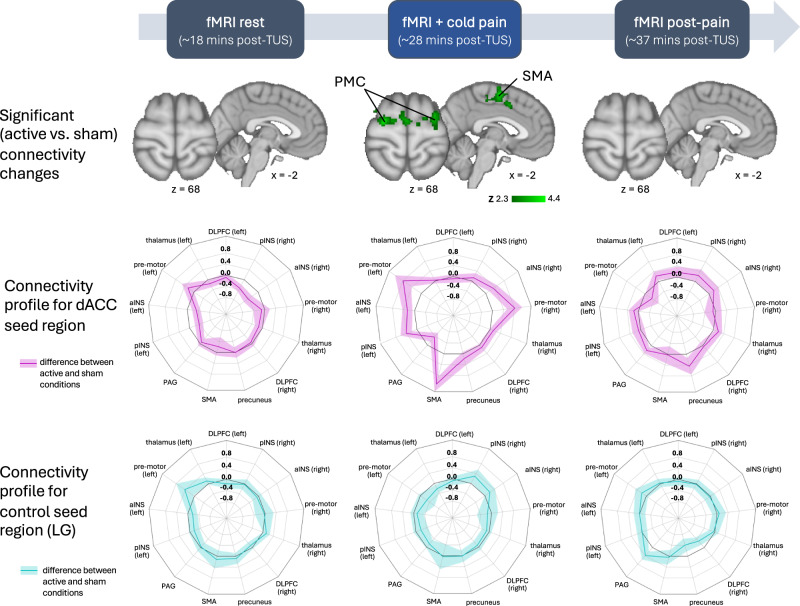
State dependency of TUS effects (*N* = 29 participants). Significant differences for the active vs. sham comparison in the whole-brain seed-based functional connectivity analysis with the dACC seed for fMRI block 1 (rest; left), fMRI block 2 (tonic cold pain; centre) and fMRI block 3 (post-pain; right) are shown in the top row. There was no significant difference during block 1 or block 3, with altered connectivity between the dACC and SMA and pre-motor cortices during block 2 (tonic cold pain). Radar plots for the difference between active and sham conditions for the dACC connectivity profile are shown in the middle row to illustrate this, with the difference close to zero in block 1 and block 3, and deviations from zero, showing differences between active and sham conditions, shown during block 2 (tonic cold pain). Finally, in the bottom row, the same radar plots are shown for a control seed region (lingual gyrus; LG), illustrating that the differences between the active and sham conditions seen with the dACC seed in the tonic pain condition are specific to TUS and not due to a general session effect. Source data are provided as a Source Data file. Error bars show the SEM. DLPFC dorsolateral prefrontal cortex, pINS posterior insular cortex, aINS anterior insular cortex, SMA supplementary motor cortex, PMC pre-motor cortex, PAG periaqueductal grey.

In addition to the tonic cold pain block, whole-brain seed-based functional connectivity analyses with the dACC seed region were conducted across the two additional fMRI blocks, at rest (before the tonic cold pain) and post-pain. There was no significant difference in functional connectivity with the dACC during either of these fMRI blocks (whole brain, mixed effects analysis, *Z* > 2.3, *p* < 0.05 comparing active vs. sham conditions), as shown in Fig. 5. This is in contrast to the tonic cold pain block, which did show significant differences in functional connectivity between the dACC and the SMA and pre-motor cortices, previously shown in Fig. 3 and shown below in Fig. 5 (centre) for completeness. The TUS-induced changes in functional connectivity seen only in the tonic pain condition and not in either of the non-pain blocks may indicate that TUS effects on dACC activity are dependent on the activation state of the region. Similar whole-brain seed-based functional connectivity analyses were also completed for a control seed-region (LG, as above) and showed that there was no significant difference in functional connectivity with the LG at any of the three fMRI blocks (whole brain, mixed effects analysis, *Z *> 2.3, *p* < 0.05 comparing active vs. sham conditions).

### Metabolite concentration results

**Fig. 6:**
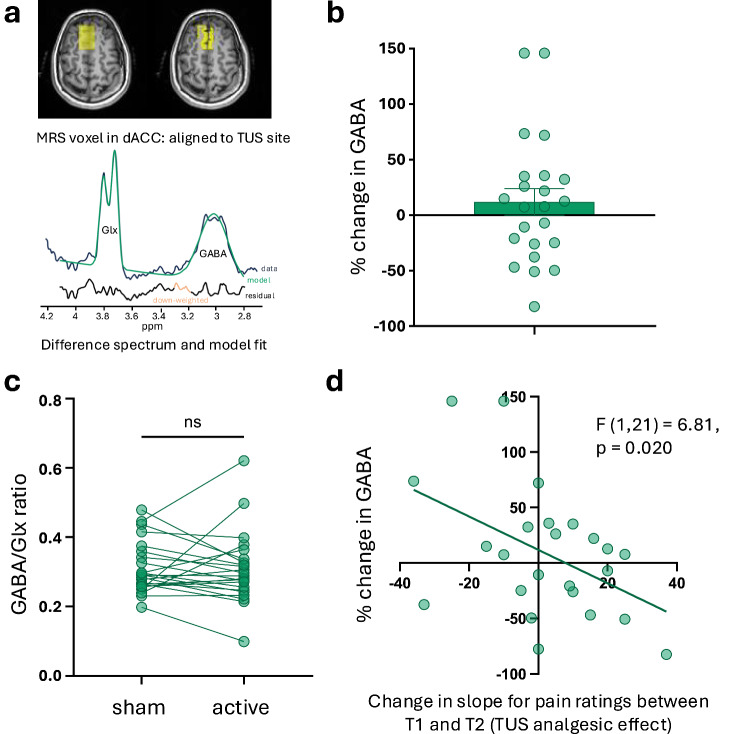
Metabolite concentration in the dACC (*N* = 23 participants). **a** Representative data from one participant showing the position of the MRS voxel over the dACC (top panel) and the difference spectrum and model fit (bottom panel), with the GABA spectrum in blue, model fit in red, and residuals shown below in black. **b** Percentage change in GABA concentration from the sham session to the active session plotted for all participants. Error bars show the SEM. Source data are provided as a Source Data file. **c** GABA/Glx ratio plotted for all participants for the sham and active sessions. Linear mixed effects modelling showed there was no significant effect of session (active or sham TUS). Fixed effects were tested using two-tailed tests, and no correction for multiple comparisons was applied, as all predictors (TUS condition, age and sex) were evaluated within a single model. Source data are provided as a Source Data file. **d** Plot showing significant negative relationship between the percentage change in GABA concentration between sessions and the change in slope for pain intensity ratings in the active and sham conditions between T1 and T2 time points – representing the analgesic effect of TUS (simple linear regression; *F*(1, 21) = 6.81, *p* = 0.02, *β* = −1.53, 95% CI [ − 2.76, − 0.31]). Source data are provided as a Source Data file.

MRS data were collected at ~55 min post-TUS to assess changes in gamma-aminobutyric acid (GABA) and glutamate (Glx) concentrations. Representative data from one participant is shown in Fig. 6a. Linear mixed effects modelling showed that there were no significant effects of session (active or sham TUS), sex or age for the concentration of GABA, Glx or the GABA/Glx ratio. The percentage change in GABA concentration between the sham and active TUS sessions is shown in Fig. 6b, illustrating there is no clear directional trend, and the GABA/Glx ratio is plotted for sham and active sessions in Fig. 6c. There was a statistically significant relationship between the percentage change in GABA concentration and the change in slope for pain intensity ratings between the T1 and T2 time points (simple linear regression; *F*(1, 21) = 6.81, *p* = 0.02, *β* = −1.53, 95% CI [ − 2.76, −0.31]), shown in Fig. 6d. This relationship indicates that for participants where active TUS had a stronger excitatory effect on the dACC, resulting in a larger decrease in GABA concentration, there was a greater analgesic effect of TUS between T1 and T2 (based on a greater change in slope of pain ratings).

### Safety profile of TUS stimulation

The stimulation was well tolerated by all participants. At the end of each session, participants completed a symptom report questionnaire, which asked them to rate the intensity of a list of symptoms they may have experienced since the stimulation. After active TUS, participants reported the following symptoms: headache (*n* = 2), scalp sensations (*n* = 1), itchiness (*n* = 1), double vision (*n *= 1), twitching (*n* = 2), balance problems (*n* = 1), changes in hand movement (*n* = 1), tingling sensations (*n* = 4), muscle tightness (*n* = 1), unusual feelings (*n* = 1), anxiety (*n* = 1), sleepiness (*n* = 3), dizziness (*n* = 3). All of these were reported as mild (defined as ‘present but not bothersome’) except for one report of sleepiness, which was reported as moderate (defined as ‘tolerable—required some intervention/medication but did not interfere with day-to-day activities’). After the sham session, participants reported: headache (*n* = 3), neck pain (*n* = 3), scalp sensations (*n* = 1), itchiness (*n* = 1), double vision (*n* = 1), twitching (*n* = 5), balance problems (*n* = 1), changes in hand movement (*n* = 2), tingling sensations (*n* = 4), unusual feelings (*n* = 1), anxiety (*n* = 2), sleepiness (*n* = 3), dizziness (*n* = 1). All of these symptoms were reported as mild. No symptoms across either condition were reported as severe. Participants did note that tingling sensations and dizziness symptoms were likely due to the tonic cold stimulus and standing up after lying in the MRI scanner, respectively. Most of the reported symptoms were rated as unrelated or unlikely to be related to the stimulation.

## Discussion

This study provides evidence that multi-focal TUS applied to dACC can modulate pain-related neural processing in healthy participants, even in the absence of significant immediate changes in subjective pain intensity. While no significant overall effect on pain ratings was observed between active and sham conditions at any single time point, there was a significantly greater reduction in pain ratings from ~28 min to ~55 min post-stimulation in the active TUS condition, suggesting a potential delayed analgesic effect. In parallel, altered sensory encoding was observed, with the typical relationship between cold temperature and pain intensity disrupted following active TUS. These behavioural findings were accompanied by significant changes in functional connectivity patterns, with increased coupling between the dACC and regions implicated in pain modulation and motor planning, including the supplementary motor area, pre-motor cortex, and anterior insular cortex, as well as decreased connectivity with PAG, a key hub in descending pain inhibition. Together, these findings suggest that TUS to the dACC can induce reorganisation within both cortical and subcortical pain networks, offering mechanistic insight into its potential utility as a non-invasive neuromodulatory intervention for pain treatment. The significant negative relationship between the percentage change in GABA concentration and the analgesic effect of TUS indicates that acute shifts in the neurochemistry balance within the dACC may underpin TUS effects, with participants having the largest decrease in GABA reporting the largest decrease in pain towards the last time point of the session.

Additional control analyses demonstrated that altered functional connectivity effects were specific to the dACC and not seen with other regions of the brain. Taken together, these findings demonstrate that the effects on connectivity fingerprints cannot be attributed to general session differences or a global influence of ultrasound on the entire brain. Instead, they provide evidence that the changes in connectivity patterns are spatially specific to the dACC and therefore most parsimoniously explained by local TUS effects. Further, the changes in functional connectivity were also shown to be specific to the pain condition only and not seen during either the baseline resting state block or the post-pain recovery block. This finding is aligned with previous results suggesting a state-dependency of TUS effects^4^, and more broadly consistent with wider evidence for state-dependent mechanisms of neurostimulation techniques^21^.

Broadly, the findings of this study showing altered neural processing following TUS are aligned with previous literature demonstrating neuromodulatory effects of TUS applied to the dACC^4,22^. In addition, the finding that TUS to the dACC resulted in altered pain processing is also aligned with previous studies demonstrating modulation of pain responses^7,12^. More specifically, the two previous studies investigating TUS to the dACC for pain modulation had quite different study designs from our study, with the first investigating effects of online TUS on heat pain in healthy participants^7^ and the second investigating effects of offline TUS on reported pain in patients with chronic pain conditions^12^, making direct comparisons of the results challenging. Both studies reported significant reductions in pain ratings overall, which was not replicated in the current study. This could be due to differences in the type of pain being assessed or differences in the TUS intervention delivered. Tonic stimuli, such as the cold pain applied here, are more temporally complex and have been shown to be differentially encoded in the brain compared to acute/phasic stimuli, relying more on emotional and evaluative circuits^23^. In addition, although experimental tonic pain has more similar network-level representations to clinical pain^24^, the fact that TUS was delivered for much longer and with different parameters may have resulted in the differential effects on pain intensity ratings between our results and those of Riis et al. More broadly, the TUS parameters and brain targets used across pain studies vary, and the optimisation of TUS parameters for eliciting maximal TUS effects is an area of ongoing research. It is likely that different stimulation parameters could have resulted in stronger analgesic effects. Ongoing work in the field aims to support evidence-based parameter selection, although gaps remain in identifying optimal frequencies for pulsed protocols and optimal duty cycles^25^.

The neuroimaging results from this study show altered connectivity between the dACC and motor-related regions, highly connected to the primary motor cortex (the pre-motor cortex and supplementary motor area). Recent neuroimaging research has identified the somato-cognitive action network (SCAN); a set of inter-effector regions within the primary motor cortex shown to be activated during action planning and highly connected to the salience network (which includes the dACC and is key for pain processing). Personalised and precision targeting of the SCAN and salience network using direct cortical stimulation (DCS) has been shown to be effective in reducing pain for patients with intractable chronic pain^26,27^, and repetitive transcranial magnetic stimulation (rTMS) targeting the motor cortex reduced pain intensity in patients with neuropathic pain^28^. In this context, our neuroimaging findings contribute to the growing body of evidence for the application of targeted neuromodulation techniques to regions associated with the SCAN and salience networks.

The role of the dACC in pain processing has been well characterised in previous studies, with a key role in the affective component of pain responses, relating to aversiveness or unpleasantness associated with the pain stimulus^29–32^. The VAS for pain intensity used in many studies, including this one, relates primarily to the sensory-discriminative aspect of pain, but since the dACC is more involved in the emotional experience of pain, outcome measures such as pain unpleasantness, mood and emotional responses may have been more sensitive for assessment of TUS effects on this brain region^9^. This is reflected in the DBS literature, with multiple studies reporting that DBS to the dACC for chronic pain results in significant improvements in quality of life but not in VAS pain intensity ratings^10,33^, although longer-term studies do show a significant reduction in numerical pain ratings at 6 months post-surgery^11,34^.

The greater decrease in active vs. sham conditions between T1 and T2 (potentially showing an analgesic effect at 55 min post-TUS) may indicate that TUS effects took longer to develop than anticipated, and if another later time point were included in the design (e.g., 1 h or more post-TUS), the TUS effects may have been stronger. Data from a macaque study conducted to investigate the temporal dynamics of TUS showed significantly altered functional connectivity between 43 min and 62 min post stimulation, with additional measures of intrinsic brain function showing effects up to 100 min (the last time point recorded)^35^. This is aligned to a study in healthy humans showing changes in functional connectivity shown across a larger number of regions at 46 min post-TUS compared to 13 min post-TUS, and significant changes in salience network and default mode network connectivity between sham and active TUS conditions at 46 min only (but not at 13 min)^4^. Overall, there are varying reports on the duration of TUS effects, with some studies reporting far longer-term effects, and wide variability in the measures used. For example, effects of TUS on reported pain intensity have been reported up to 7 days post-stimulation in chronic pain patients^12^, and in vitro research has shown that enhanced neuronal excitability in primary rat cortical neurons lasts up to 8 h^36^. Further research is needed to understand the temporal dynamics of the effects of offline TUS, and to elucidate the neural mechanisms underpinning these effects across short-term (minutes), mid-term (hours) and long-term (days) timescales. Since there was no overall main effect of condition (active or sham TUS) and the statistical significance for the decrease between T1 and T2 is marginal (*p* = 0.043), the potential delayed analgesic effect discussed here requires replication in further studies. There were greater shifts in pain intensity ratings over time in the active TUS condition, with a greater increase between T0 and T1 (although not significantly different) and a greater decrease between T1 and T2, compared to sham. This could be explained in a framework of neuroplasticity, potentially reflecting TUS-induced sensitisation at the start, shifting to adaptation and pain inhibition over time. Future work is needed to explore these temporal dynamics of TUS effects further.

The study did have several limitations. The sample size is relatively small, with 29 participants for fMRI and 23 for MRS, limiting the statistical power of analyses and potentially impacting generalisability of the findings. In relation to the fMRI data, although a multi-echo acquisition was used, the final analysis was limited to data from a single echo time due to excessive noise in the combined data. As a result, we were unable to take advantage of the potential benefits of multi-echo processing, such as improved signal-to-noise ratio. Furthermore, the selected echo time was not specifically optimised for use in our analysis, which may have affected sensitivity. However, given the substantial noise in the combined data, using the second echo alone provided a more reliable and interpretable dataset. It is also worth noting that the scanner software update, which was completed in the middle of data collection, necessitated a change in the scan sequence, which may have introduced additional variability in data quality. The relatively short scan duration of 6.5 min was required to ensure tolerability of the tonic cold stimulus for the length of the scan block; however, this is a limitation, with recent studies demonstrating that longer scans improved reliability in brain-wide association studies^37^.

A strength of the study was the double-blinded design, which aimed to reduce any bias arising from both participants and researchers towards active vs. sham stimulation. Blinding effectiveness was assessed for the researcher administering the stimulation in a sample of sessions (*n* = 34 sessions), with exactly 50% accuracy rate, indicating the blinding procedures in place for researchers were very effective. For participants, they were not informed prior to completion of the study that there was a ‘sham’ session, and the sound delivered via bone-conducting headphones during the sham session aimed to closely match the active TUS. However, blinding effectiveness was not assessed. Assessment of blinding effectiveness for participants would have been valuable to evaluate any bias potentially introduced by participants’ expectations. The researcher conducting data analysis was unblinded at that stage, which could also be considered a limitation of the study, as it could have introduced bias.

Overall, this study provides mechanistic insight into how multifocal TUS targeting the dACC can alter pain-related brain function in healthy individuals. Together, these findings demonstrate the capacity of TUS to influence distributed brain circuits involved in the complex experience of pain, supporting its potential as a targeted, non-invasive intervention. By demonstrating this network-level modulation in regions implicated in chronic pain, this work provides a crucial mechanistic bridge supporting the translation of dACC-TUS into clinical trials for persistent pain conditions. Characterising the specific changes in dACC-centred network dynamics highlights potential biomarkers and therapeutic targets that could guide target optimisation and patient selection in future translational studies of TUS for chronic pain. Additionally, these results highlight the importance of including longer post-stimulation assessment periods in future studies, as well as expanding outcome measures to better capture the affective and evaluative dimensions of pain processing.

In conclusion, this study extends current understanding of the effects of TUS on the dACC by demonstrating functionally meaningful changes in pain perception and brain connectivity. The findings lay important groundwork for future translational research exploring the therapeutic use of TUS in chronic pain populations. Further work in larger samples, using multimodal approaches and longer follow-up periods, will be critical to fully characterise the efficacy, duration, and mechanisms of TUS-induced modulation of pain.

## Methods

### Participants and approvals

The study was approved by the University of Plymouth Faculty of Health Staff Research Ethics and Integrity Committee (reference: 4183). 35 healthy participants were recruited to take part in the study. Exclusion criteria were applied for TUS and MRI safety and to ensure participants were healthy, with no pain-related conditions. Two participants were excluded after the first visit due to previously experiencing a severe concussion (*n* = 1), a contraindication for TUS, and due to an incidental finding in the first MRI (*n* = 1). Therefore, 33 participants completed the study (mean age 26.3 ± 10.2 years, range 21-66, 19 female, 14 male, sex and gender aligned by self-report). All participants gave written informed consent prior to taking part. Participants were compensated £80 for completing all 3 study sessions. All sessions took place at the Brain Research and Imaging Centre in Plymouth, United Kingdom.

### Study design

The study design was a double-blind, randomised crossover study in healthy participants. The study consisted of three visits: the first to acquire T1-weighted and PETRA images for acoustic simulations, followed by two main TUS-fMRI sessions with either active TUS or sham (Fig. 1a). At each main session, first active/sham TUS was applied during a tonic cold stimulus, then an MRI scan was completed with fMRI blocks during baseline, tonic cold and recovery, and a magnetic resonance spectroscopy (MRS) block during tonic cold.

### Tonic pain model

Tonic pain was induced using a modified cold pressor test^38,39^ with gelled water (temp range: 3.9 ± 1.8 °C). The gelled water was prepared in a 2 L plastic container using water (1.6 L), a thickening product containing modified cornstarch (99 g), and salt (100 g), kept in the freezer for 12 h prior to use^40^. Verbal pain intensity ratings were collected after each of the three tonic cold pain stimuli, on a numerical rating scale (NRS) from 0—no pain, to 100—worst pain imaginable.

### TUS stimulation protocol

The NeuroFUS transducer power output (TPO) system and CTX-500-4 transducer (Brainbox Ltd., Cardiff, UK), a four-element annular transducer (diameter = 64 mm, central frequency = 500 kHz, with steering range between 27.3 and 82.6 mm), was used to deliver the TUS protocol. The dACC targeting and optimal transducer placement were individually planned using structural MRI scans (T1-weighted and PETRA) and k-Plan software (BrainBox, Inc.) to run acoustic and thermal simulations for each participant, to ensure accurate pressure delivery at the target area and adherence to safety protocols. A multi-site approach, with 3 sites within the dACC, was used in order to maximise target engagement and subsequently the neuromodulatory effect, since the dACC is a relatively large region compared to the focal size of the TUS beam. The three dACC targets were defined anatomically using MNI co-ordinates: target A: −3,37,12; target B: −3,33,17; and target C: −3,28,22. Repetitive TUS followed a 10 Hz-patterned protocol with a 10% duty cycle (fundamental frequency = 500 KHz, pulse duration = 10 ms, pulsed every 100 ms), applied for 80 s at each target sequentially (4 min in total), in the same order from A to C in all participants. We opted for this 10 Hz, 10% duty-cycle protocol to produce inhibitory neuromodulation of the dACC, based on its alignment with alpha-frequency inhibitory dynamics and prior evidence of inhibitory or analgesic effects^6,41–43^. The I_SPPA_ in water was maintained at 54 W/cm² across participants; with this higher input ISPPA used to account for skull attenuation, which was modelled in the *k*-Plan acoustic simulations, to ensure safety and to assess the ISPPA reaching the brain target. We adhered to safety guidelines for human ultrasound exposure as defined by the International Thermal and Radiological Ultrasound Safety Standards and Thresholds (ITRUSST^20^). A full description of the stimulation parameters aligned to the ITRUSST consensus on standardised reporting for TUS^44^ is included in Supplementary Table 2. Additionally, a table including the stimulation parameters from three other pain TUS studies is included in Supplementary Table 3 to enable comparison of the protocols used.

To ensure ultrasound transmission, a layer of ultrasound gel (Aquasonic 100, Parker Laboratories Inc.) was applied at the transducer placement site, with a 2 cm gel pad (Aquaflex, Parker Laboratories Inc.) positioned between the transducer and the participant’s head. Hair was not shaved; while applying the layer of gel, the hair was carefully combed and smoothed to eliminate air gaps. Neuronavigation was performed using Brainsight v2.5 (Rogue Research Inc., Montréal, Québec, Canada) with T1-weighted anatomical MR images. During each session, focal depth measurements to each target obtained from Brainsight were entered into the NeuroFUS TPO system before stimulation, and the trajectory was sampled to support post-session confirmatory acoustic simulations.

The sham condition consisted of a matched auditory stimulus delivered to participants via bone-conducted headphones. Since the study was double-blind, all procedures were matched at both sessions (with the same neuro-navigation set-up and transducer placement at each target). An independent researcher managed the blinding and administered either active or sham TUS depending on the randomisation for each participant. Data were unblinded prior to analysis.

### Imaging data acquisition (MRI and MRS)

Data were collected on a Siemens MAGNETOM Prisma 3 T scanner with a 64-channel head coil. At visit 1, a T1-weighted structural scan and a pointwise encoding time reduction with radial acquisition (PETRA) scan were collected for use in acoustic simulations and neuronavigation. The T1-weighted structural scan was acquired with an MPRAGE sequence with a TR of 2.1 s, echo time of 2.26 ms, inversion time of 900 ms, flip angle of 8°, GRAPPA acceleration factor of 2, matrix size of 256×256, 176 slices, and with 1 mm³ isotropic voxels. The PETRA was acquired with a TR of 3.61 ms, an echo time of 0.07 ms, a flip angle of 8°, 320 slices per slab and a slice thickness of 0.75mm. 3D distortion correction was applied following acquisition. The PETRA scan was converted to a pseudo-CT scan for use in acoustic simulations using the PETRA-to-CT MATLAB toolbox^45^.

At visits 2 and 3, three resting-state BOLD scans were completed (rest/baseline, tonic cold pain, and post-pain recovery), followed by a field map and T1-weighted structural scan, and finally a magnetic resonance spectroscopy (MRS) scan. Due to an upgrade of the scanner software, the second 14 datasets were collected with a slightly modified scan sequence than the first 15 (for BOLD and MRS scans only). Modified parameters are indicated in square brackets following the initial parameter used, if applicable. Resting-state BOLD fMRI data were acquired using a multi-echo echo-planar imaging (EPI) sequence with repetition time (TR) of 1.5 s [1.55 s], 4 echo times (11.0, 27.25, 43.5, and 59.75 ms [13.6, 29.86, 46.1 and 62.36 ms]), flip angle of 77°, 2.6 mm³ isotropic voxels, 51 axial slices, interleaved acquisition, 220 mm field of view, matrix size of 84×84, 2.6 mm slice thickness, GRAPPA acceleration factor of 2, multi-band acceleration factor of 3, bandwidth of 2480 Hz/Px, and 240 volumes per run. The field map was acquired to enable correction of field inhomogeneity during analysis, with 2.6 mm^3^ isotropic voxels and a 220 mm field of view. The T1-weighted scan had the same parameters as for visit 1. MRS data were acquired in a 3.5×2.5×2.5 cm^3^ voxel positioned in the dACC (TUS target region) using a MEGA-PRESS sequence to quantify GABA concentrations, with TR of 2 s, echo time of 68 ms, excitation flip angle of 90°, and 256 [128] averages. Water suppression was achieved using a frequency-selective saturation pulse (bandwidth = 35 Hz) [VAPOUR (bandwidth = 60 Hz)], and MEGA editing pulses were centred at 1.9 ppm and 7.5 ppm to detect GABA, with editing pulse flip angle of 180°. Spectral data were collected with a vector size of 2048, bandwidth of 2000 Hz [1850 Hz], and acquisition duration of 1024 ms [1107 ms]. A water unsuppressed reference was collected with 8 averages. Automatic frequency and shim adjustments were applied prior to acquisition.

### Post-TUS symptom questionnaire

After each session, participants were asked to complete a symptom questionnaire, which consisted of a list of symptoms for which they were asked to rate the intensity they experienced the symptom using a 4-point scale (absent, mild, moderate, severe) and whether they thought their experience was related to the stimulation on a 5-point scale (unrelated, unlikely, possible, probable, definite). Participants could note up to two additional symptoms and could provide comments. See Supplementary Fig. 1 for the full questionnaire.

### Statistical analysis

For behavioural data, the relationship between pain intensity ratings and TUS condition (as well as additional potentially confounding factors) was assessed using linear mixed effects modelling and robust linear mixed effects modelling in R v4.4.0. The following general linear model (GLM) was used:

rating ~ TUS condition * time point * gel temperature + sex + session order + age + (1 | subject)

The model either included all time points (for primary analysis) or included only T1 and T2 for the exploratory analysis of effects occurring during this time frame. The relationship between pain intensity rating and temperature of the tonic cold stimulus in both the active and sham TUS sessions was assessed using simple linear regression in GraphPad PRISM v10.1.0. To assess whether these relationships were driven by outliers, the analysis was also repeated using robust regression implemented in MATLAB v25.2.0.

Resting state fMRI data were analysed using tools in FMRIB Software Library v6.0 (FSL)^46–48^. Structural and magnitude images were brain extracted^49^ and a calibrated field map image was prepared as required for B0 unwarping. Due to excessive noise in the multi-echo data, only the second echo time (TE2; 27.25 [29.86]) was used for analysis of the functional data. Pre-processing steps, including registration to structural and MNI standard images, B0 unwarping, motion correction, spatial smoothing (5 mm) and high-pass temporal filtering, were conducted using FEAT^50^. For seed-based analysis, the time course of BOLD activity from the seed region was extracted and used to generate individual statistical maps of the functionally correlated activity across the whole brain for each participant, using the GLM approach implemented with FEAT^50^. Motion outliers identified using the fslmotionoutliers tool, average signal from the white matter and cerebrospinal fluid generated using anatomical segmentations for each tissue type, and the six motion parameters from the motion correction step were included as nuisance covariates. Group-level whole-brain, mixed-effects analysis with a cluster-based correction for multiple comparisons was performed using FEAT to search for differences in functional connectivity between the active TUS and sham TUS conditions^51^. The mask for the dACC seed-region was generated by creating 5 mm spheres around each of the 3 targets, combining these, and then warping this mask to each individual participant’s functional space. For each participant, any regions of the dACC mask that overlapped with thresholded white matter or cerebrospinal fluid masks were subtracted to create an individualised seed in grey matter only. To explore changes in resting state network connectivity, probabilistic independent component analysis (ICA) as implemented in MELODIC was used^52–54^. Data from both active and sham TUS sessions were pre-processed by masking non-brain voxels, applying voxel-wise demeaning and normalisation of the voxel-wise variance, then projected into a 20-dimensional subspace using principal component analysis. Spatial maps from this group-average analysis were used to generate subject-specific versions and associated timeseries, using dual regression^55,56^. Group differences between the active TUS and sham TUS conditions were then compared using randomise with threshold-free cluster enhancement (TFCE; 5000 permutations, *p* < 0.05)^57^. These two approaches offer different strengths, with seed-based analysis enabling exploration of targeted hypotheses about connectivity between the dACC seed and other brain regions, whereas the ICA-based analysis enables data-driven, whole-network exploration without prior assumptions such as a predefined seed region.

To assess whether the observed effects on the connectivity fingerprint of the dACC during pain were specific to the dACC and only attributable to TUS, or instead reflected a general session effect, we conducted a control analysis. We identified brain regions that were least functionally connected to the dACC under the sham condition. For each region, we computed the connectivity fingerprints separately for the active TUS and sham sessions by generating matrices of covariation between the voxels in this region and an a priori defined constellation of regions used in the main pain rsfMRI analyses (Fig. 3c). Pairwise distances between all elements of these matrices were then calculated using the cityblock (Manhattan) distance metric, providing a measure of dissimilarity between session-specific connectivity profiles. This analysis, in theory, should reveal where in the brain the TUS effects were maximum. One can imagine that this should be mainly true where TUS has been applied, e.g., in the dACC and not the control sites. In addition, a further group-level whole-brain, mixed-effects analysis with a cluster-based correction for multiple comparisons was performed using FEAT to search for differences in functional connectivity with a control region between the active TUS and sham TUS conditions^51^. The control seed-region was the LG with the mask for the seed-region generated by creating 3 5 mm spheres within the LG grey matter, and combining these, resulting in a mask of similar dimensions to the main dACC mask used.

For MRS, data were analysed using the MATLAB-based Gannet 3.4.0 toolkit^58^. Processing steps included frequency and phase correction conducted with robust spectral registration^59^, 3 Hz exponential line broadening, and individual transient averaging. The GABA and Glx peaks at 3.0ppm and 3.75 ppm, respectively, were modelled relative to water and corrected for voxel tissue composition using segmentation of T1-weighted structural images conducted with SPM^13,60^. Data quality was assessed via visual inspection for spectral artefacts and on quality metrics including FWHM, GABA+ signal to noise ratio (SNR) and model fit error, resulting in 3 datasets being excluded from analysis. One further dataset was excluded as an outlier, identified using the ROUT method (*Q* = 1%) in GraphPad PRISM, resulting in 23 datasets included for analysis (mean age 26.6 ± 11.3 years, range 21-66, 14 female, 9 male). The relationship between metabolite concentrations (GABA, GLx and GABA/Glx ratio) and TUS condition (as well as additional potentially confounding factors) was assessed using linear mixed effects modelling in R v4.4.0. The following GLM was used for this:

metabolite concentration ~ TUS condition + sex + age + (1 | subject)

The relationship between the percentage change in GABA concentration between sessions and the change in slope for pain intensity ratings in the active and sham conditions between T1 and T2 time points was assessed using simple linear regression in GraphPad PRISM v10.1.0.

### Reporting summary

Further information on research design is available in the Nature Portfolio Reporting Summary linked to this article.

## Supplementary information

Supplementary Information

Reporting Summary

Transparent Peer Review file

## Source data

Source Data

## Data Availability

The behavioural, MRI and MRS data generated in this study are available on the Open Science Framework (OSF) at https://osf.io/vp2gz/. The data used to generate all figures in the paper are provided in the Source Data file. Source data are provided with this paper.

## Code availability

The code for running behavioural and neuroimaging data analysis presented in this study is also available at https://osf.io/vp2gz/.
