# Supplementary material for: Multi-focal ultrasound neuromodulation to the dorsal anterior cingulate cortex disrupts behavioural and neural pain processing
Source: Nat Commun. 2026 May 9;17:6269. doi: 10.1038/s41467-026-72934-3 (PMC13376748; doi:10.1038/s41467-026-72934-3)
Supplement: Supplementary file 1 — Supplementary Information [file 41467_2026_72934_MOESM1_ESM.pdf]

SUPPLEMENTARY MATERIALS

Supplementary Table 1: Individual post-stimulation simulation results.

Participant	Intensity (I_{SPPA}) in dACC			Pressure (kPa) in dACC			Thermal dose in soft tissues (CEM43°C)			Mechanical index in soft tissues (MI)		
	A	B	C	A	B	C	A	B	C	A	B	C
1	2.85	2.09	4.36	324.89	269.74	391.89	0.001	0.002	0.003	0.40	0.36	0.50
2	2.83	2.96	3.51	301.01	306.88	352.95	0.001	0.002	0.003	0.40	0.42	0.44
3	1.51	2.41	5.29	230.88	289.45	443.00	0.003	0.005	0.010	0.73	0.87	0.80
4	2.88	3.08	3.94	325.93	336.46	364.07	0.004	0.009	0.012	0.86	0.92	1.56
5	4.18	5.40	5.66	392.47	437.13	455.67	0.005	0.010	0.015	1.00	0.79	0.86
6	1.77	5.44	6.32	257.96	433.93	475.64	0.002	0.003	0.006	0.70	0.70	0.71
7	7.04	6.11	5.61	495.60	466.31	448.07	0.003	0.004	0.008	0.83	0.75	0.86
8	3.27	2.96	5.27	347.82	318.48	419.19	0.003	0.009	0.012	0.80	0.74	0.70
9	4.61	5.31	5.42	417.72	453.10	440.30	0.002	0.004	0.006	0.57	0.65	0.77
10	5.28	5.29	5.05	434.98	434.41	408.89	0.002	0.004	0.007	0.77	0.70	0.73
11	2.47	2.45	3.24	297.54	280.90	348.51	0.005	0.009	0.015	0.69	1.07	1.02
12	3.07	5.01	5.25	328.56	409.87	438.71	0.002	0.004	0.005	0.74	0.75	0.86
13	2.05	4.18	6.03	275.37	370.41	460.00	0.002	0.004	0.007	0.66	0.55	0.59
14	0.52	0.54	5.56	138.29	140.23	436.37	0.003	0.009	0.012	0.63	0.65	0.99
15	5.89	5.39	5.11	452.77	440.09	428.83	0.002	0.007	0.009	0.72	0.71	0.86
16	3.05	4.60	5.81	324.19	400.38	455.95	0.002	0.006	0.013	0.67	0.70	1.00
17	1.61	2.16	3.24	245.08	281.19	325.95	0.002	0.005	0.008	0.56	0.63	0.66
18	1.39	1.39	1.93	222.26	227.24	263.20	0.002	0.007	0.012	0.67	0.82	1.00
19	2.36	2.05	3.55	290.67	271.99	358.04	0.002	0.006	0.012	0.73	0.77	1.15
20	0.95	1.55	2.14	182.20	238.80	271.71	0.002	0.007	0.009	0.74	0.79	0.84
21	7.73	6.87	4.57	513.32	481.16	407.82	0.002	0.009	0.016	0.86	0.99	1.14
22	2.06	2.34	3.21	263.06	288.15	330.84	0.003	0.011	0.021	0.75	1.00	1.08
23	2.89	3.11	2.45	327.72	331.35	292.29	0.003	0.007	0.011	0.70	0.64	0.84
24	2.96	2.91	2.71	328.76	319.75	315.04	0.002	0.006	0.010	0.78	0.63	0.75
25	4.06	5.08	3.50	381.40	427.61	351.94	0.002	0.006	0.011	0.63	0.70	0.69
26	4.69	3.69	4.53	404.10	344.64	419.09	0.003	0.013	0.023	0.89	0.97	0.76
27	5.16	5.22	7.27	433.54	408.61	500.96	0.002	0.006	0.012	0.72	0.74	1.15
28	2.56	2.43	1.98	304.72	296.65	266.94	0.002	0.006	0.010	0.65	0.83	0.70
29	2.80	2.86	3.32	323.47	316.43	352.58	0.002	0.006	0.012	0.55	0.61	0.73
30	3.23	3.88	3.79	348.91	354.64	363.48	0.003	0.026	0.036	0.90	1.14	1.04
31	4.73	6.74	6.71	402.30	477.72	496.89	0.002	0.005	0.010	0.63	0.78	0.73
32	1.53	1.47	2.50	236.99	223.78	298.74	0.002	0.006	0.012	0.92	0.78	0.95

Table includes the post-stimulation simulation results for each participant for the intensity (I_{SPPA}) in the dorsal anterior cingulate cortex (dACC), the pressure (kPA) in the dACC, the thermal dose in soft tissues (CEM43°C) and the mechanical index in soft tissues (MI).

Supplementary Table 2: TUS protocol as per the ITRUSST consensus on standardised reporting.

Transducer and drive system description	
Transducer manufacturer and model number	Sonic Concepts NeuroFUS CTX-500-4
Transducer centre frequency	500kHz
Transducer geometry	60mm diameter x 64mm radius of curvature
Drive system components, including manufacturer and model number (e.g., signal generator and amplifier or integrated driving system)	Sonic Concepts Transducer Power Output (TPO)
Drive system settings	
Operating frequency	500kHz
Output level settings	ISPPA = 54W/cm2
Focal position settings	Individualised for each participant
Description of transducer coupling method	Layer of ultrasound gel *(Aquasonic 100, Parker Laboratories Inc.)* was applied at the transducer placement site, with a 2cm gel pad *(Aquaflex, Parker Laboratories Inc.)* positioned between the transducer and the participant's head. Hair was not shaved; while applying the layer of gel the hair was smoothed to eliminate air gaps.
Free field acoustic parameters	
Reference position for measurements	Transducer exit plane
Spatial-peak pressure amplitude	1.27mPa
Position of spatial-peak pressure amplitude (relative to reference position)	57.6mm
Size of focal volume (−3 dB and −6 dB axial and lateral widths)	Focal volume at -3 dB (50%): 129.59 mm^3<br>Focal volume at -6 dB (25%): 375.91 mm^3<br>-3 dB axial width: 16.93 mm<br>-3 dB lateral width: 4.44 mm<br>-6 dB axial width: 24.11 mm<br>-6 dB lateral width: 6.07 mm
Position of centre of focal volume (centre of −3 dB relative to reference position)	59.9mm
Description of how free field parameters were obtained (including details of measurement equipment)	Obtained using k-Plan free-field (water) simulation with ISPPA of 54W/cm^2 and focal depth of 60mm (typical for dACC)
Pulse timing parameters	

	Duration	Ramp duration	Ramp shape	Repetition interval / Frequency	Notes
Pulse	10ms	0	Rectangular	100ms / 10Hz	
Pulse train	80s	0	Rectangular		

In situ estimates of exposure parameters	
Estimated in situ spatial-peak pressure amplitude	Reported in Supplementary Table 1
Estimated in situ pressure amplitude at the target	Reported in Supplementary Table 1
Estimated in situ mechanical index	Reported in Supplementary Table 1
One of the following thermal metrics: temperature rise, thermal index, or thermal dose	Thermal dose reported in Supplementary Table 1
Description of how in situ estimates were obtained	Individual acoustic and thermal simulations conducted using structural MRI scans (T1-weighted and PETRA) and k-Plan software *(BrainBox, Inc.)*

Table shows the completed checklist for the TUS intervention used in the study, as per the ITRUSST consensus on standardised reporting for transcranial ultrasound stimulation.

Supplementary Table 3: TUS protocols for recent pain studies.

Study	Device	Design and brain target	Ff (kHz)	PRF (Hz)	Pulse duration (ms)	Duty cycle (%)	Sonication duration	Pulse repetition interval	Number of sonications	Average peak pressure (MPa)
Badran et al. 2020	BrainSonix BXPulsar 1002	Offline, targeting thalamus (MRI-guided to right anterior thalamus)	650	10	5	5%	30s	30s	10	0.72
Strohman et al. 2024	Sonic Concepts, model H-104	Online, targeting dACC (MNI: 0,18,30)	500	1000	0.36	36%	100ms	1ms	40	0.1152
Riis et al. 2024	Bespoke device with 2 phased array Transducers (see paper for details)	Offline, targeting ACC (8 ACC subregions, with 4 selected)	650	100<br>(Pulse Train RF: 1.42)	5<br>(Train duration: 30)	50%<br>(30%)	30ms<br>(3 minutes)	10ms<br>(1000ms, off period = 700ms)	12 (across 4 targets)	1
**Current study**	**NeuroFUS CTX-500-4**	**Offline, targeting dACC MNI: A: -3,37,12 B: -3,33,17 C: -3,28,22**	**500**	**10**	**10**	**10%**	**80s**	**90ms**	**3 (multi-focal)**	**0.354**

Table outlines the TUS device, study design and brain target, and the parameters for the TUS protocol used in three recent pain studies (Badran et al. 2020, Strohman et al. 2024, and Riis et al. 2024), in addition to the current study.

Ff; fundamental frequency, PRF; pulse repetition frequency

Supplementary Figure 1: Post-TUS Symptom Report Questionnaire.

Absent = Not present

Mild = Present but not bothersome
Moderate = Tolerable - required some intervention/medication, but did not interfere with day-to-day activities
Severe = Intolerable - required contact with a GP or hospital A&E
Use the middle box to specify whether you think it might be related to the stimulation.
Use the box to the right of each symptom to provide details. e.g. describe what you felt, how long it lasted, medication you took to relieve the symptoms.

**Since your stimulation, have you had...?**	**Intensity of symptom**	**Relationship to stimulation**	**Please provide details**
a headache	absent	unrelated	
neck pain	mild	unlikely	
tooth pain	moderate	possible	
unusual feelings on your head or scalp	severe	probable	
itchiness		definite	
changes to your hearing			
speech problems			
vision problems (e.g. double vision)			
unusual twitching or muscle movement			
difficulties in balance			
changes in the movement of your strongest hand			
numbness or tingling sensations			
muscle tightness of the face or arm			
unusual feelings, attitudes or emotions			
anxiety, worried thoughts or nervousness			
increased sleepiness			
changes to your sleep pattern			
difficulty paying attention			
increased forgetfulness			
nausea or sickness to the stomach			
dizziness or light-headedness			
a seizure within the last 24 hours			
other symptom:			
other symptom:			
**Do you have anything else to report?**			

Post-TUS symptom report questionnaire that participants were asked to complete after each TUS session to assess whether symptoms occurred as a result of sham or active stimulation.

Supplementary Figure 2: Temperature of cold gel stimulus for all time points pooled (far left) and for T0, T1 and T2 time points separately (N = 32 participants).

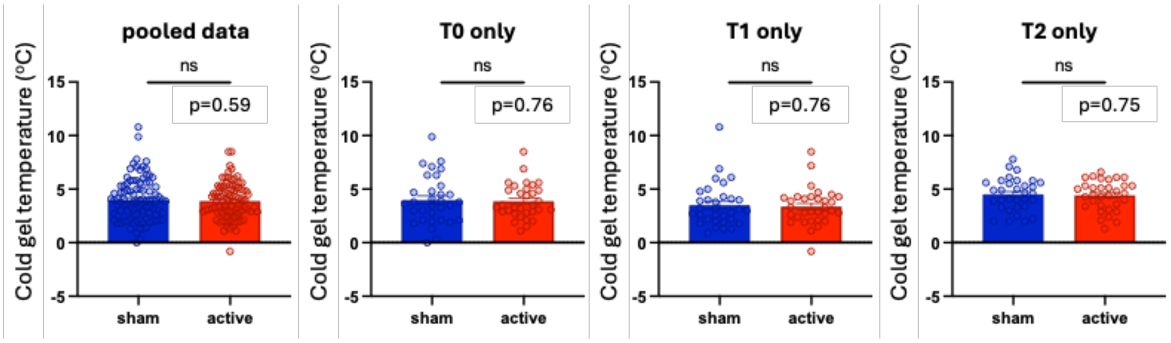

Plots show the cold gel temperature for all time points pooled and for each of T0, T1 and T2 individually. There were no significant differences between the temperature of the gel stimulus between the active and sham conditions for either all three time points pooled data (paired t-test, t(df) = −0.54, p = 0.59, R^2 = 0.003, 95% CI [−0.55, 0.31]), or for any of the time points individually (T0 paired t-test; t(df) = 0.31, p = 0.76, R^2 = 0.0031, 95% CI [−0.87, 0.64], T1 paired t-test; t(df) = 0.30, p = 0.76, R^2 = 0.0029, 95% CI [−1.04, 0.77], T2 paired t-test; t(df) = 0.32, p = 0.75, R^2 = 0.0032, 95% CI [−0.74, 0.54]).
