# Supplementary material for: Multi-focal ultrasound neuromodulation to the dorsal anterior cingulate cortex disrupts behavioural and neural pain processing
Source: Nat Commun. 2026 May 9;17:6269. doi: 10.1038/s41467-026-72934-3 (PMC13376748; doi:10.1038/s41467-026-72934-3)
Supplement: Supplementary file 2 — Reporting Summary [file 41467_2026_72934_MOESM2_ESM.pdf]

nature portfolio

Corresponding author(s): Sophie Clarke

Last updated by author(s): Apr 7, 2026

Reporting Summary

Nature Portfolio wishes to improve the reproducibility of the work that we publish. This form provides structure for consistency and transparency in reporting. For further information on Nature Portfolio policies, see our Editorial Policies and the Editorial Policy Checklist.

Statistics

For all statistical analyses, confirm that the following items are present in the figure legend, table legend, main text, or Methods section.

n/a	Confirmed	
☐	☒	The exact sample size (*n*) for each experimental group/condition, given as a discrete number and unit of measurement
☐	☒	A statement on whether measurements were taken from distinct samples or whether the same sample was measured repeatedly
☐	☒	The statistical test(s) used AND whether they are one- or two-sided<br>*Only common tests should be described solely by name; describe more complex techniques in the Methods section.*
☐	☒	A description of all covariates tested
☐	☒	A description of any assumptions or corrections, such as tests of normality and adjustment for multiple comparisons
☐	☒	A full description of the statistical parameters including central tendency (e.g. means) or other basic estimates (e.g. regression coefficient) AND variation (e.g. standard deviation) or associated estimates of uncertainty (e.g. confidence intervals)
☐	☒	For null hypothesis testing, the test statistic (e.g. *F*, *t*, *r*) with confidence intervals, effect sizes, degrees of freedom and *P* value noted<br>*Give P values as exact values whenever suitable.*
☒	☐	For Bayesian analysis, information on the choice of priors and Markov chain Monte Carlo settings
☒	☐	For hierarchical and complex designs, identification of the appropriate level for tests and full reporting of outcomes
☐	☒	Estimates of effect sizes (e.g. Cohen's *d*, Pearson's *r*), indicating how they were calculated

Our web collection on statistics for biologists contains articles on many of the points above.

Software and code

Policy information about availability of computer code

Data collection	Transcranial ultrasound stimulation was delivered using the NeuroFUS TPO and CTX-500-4 transducer (Brainbox Ltd., Cardiff, UK). Neuronavigation was performed with the Brainsight software v 2.4.11 (Rogue Research Inc., Montréal, Québec, Canada).<br>MRI scans were acquired on a Siemens MAGNETOM Prisma 3T scanner (VE11E, Siemens Healthineers, Erlangen, Germany) with a 64-channel head coil.
Data analysis	Acoustic simulations were performed using k-Plan software (Brainbox Inc.). The PETRA scan was converted to a pseudo-CT scan for use in acoustic simulations using the PETRA-to-CT MATLAB toolboxare available on GitHub: https://github.com/ucl-bug/petra-to-ct.<br>FMRI data were pre-processed and analysed using FEAT (FMRI Expert Analysis Tool) and MELODIC tools from the FMRIB Software Library v6.0 (FSL; www.fmrib.ox.ac.uk/fsl). The code for running behavioural and neuroimaging data analysis presented in this study are also available at https://osf.io/vp2gz/.

For manuscripts utilizing custom algorithms or software that are central to the research but not yet described in published literature, software must be made available to editors and reviewers. We strongly encourage code deposition in a community repository (e.g. GitHub). See the Nature Portfolio guidelines for submitting code & software for further information.

Data

Policy information about availability of data

All manuscripts must include a data availability statement. This statement should provide the following information, where applicable:

- Accession codes, unique identifiers, or web links for publicly available datasets
- A description of any restrictions on data availability
- For clinical datasets or third party data, please ensure that the statement adheres to our policy

The behavioural, MRI and MRS data generated in this study are available on the Open Science Framework (OSF) at https://osf.io/vp2gz/. The data used to generate all figures in the paper are provided in the Source Data file.

Research involving human participants, their data, or biological material

Policy information about studies with human participants or human data. See also policy information about sex, gender (identity/presentation), and sexual orientation and race, ethnicity and racism.

Reporting on sex and gender	Sex and gender information were determined based on participant self-reports. Our study included participants of all genders, and the findings are not specific to one sex or gender. In the study design, we aimed to recruit a balanced number of participants from both sexes. No sex-based analyses were performed.
Reporting on race, ethnicity, or other socially relevant groupings	The study had no focus on race, ethnicity, or other socially relevant groupings; consequently, we endeavored to collect data as inclusively as possible without gathering specific information on these demographics.
Population characteristics	33 participants completed the study (mean age 26.3±10.2 years, range 21-66, 19 female, 14 male, sex and gender aligned by self-report, with no participants identifying as non-binary). Participants were screened for contraindications to TUS and MRI, had no current diagnosis of neurological or psychiatric disorders, and were free of psychoactive medications at the time of the study.
Recruitment	Participants were recruited through advertisements posted via the University's email circulation lists and through word-of-mouth. Participants received monetary compensation for their participation in the study. Potential sources of bias include self-selection, as individuals motivated to participate in research, particularly those within the university community, may be overrepresented in the sample.
Ethics oversight	The study was approved by the University of Plymouth Faculty of Health Staff Research Ethics and Integrity Committee (reference: 4183).

Note that full information on the approval of the study protocol must also be provided in the manuscript.

Field-specific reporting

Please select the one below that is the best fit for your research. If you are not sure, read the appropriate sections before making your selection.

☒ Life sciences ☐ Behavioural & social sciences ☐ Ecological, evolutionary & environmental sciences

For a reference copy of the document with all sections, see nature.com/documents/nr-reporting-summary-flat.pdf

Life sciences study design

All studies must disclose on these points even when the disclosure is negative.

Sample size	Since there are no previous fMRI studies on the behavioral and neural effects of TUS targeted to the dACC for tonic cold pain, a formal power calculation was not possible. As this is the first fMRI investigation of this paradigm, and no prior effect size estimates were available to inform a formal power analysis, we therefore selected a sample size of 30 participants, which is consistent with sample sizes commonly used in exploratory task-based fMRI studies and reflects a balance between feasibility and the need to obtain stable within-subject activation estimates.
Data exclusions	Exclusion criteria were established before any analyses were performed. fMRI data were excluded if motion exceeded 0.25 mm (relative displacement between consecutive volumes) for more than 50% of the volumes, or if image artifacts were discovered during visual data quality inspection. Three fMRI datasets were excluded on this basis, due to artefacts.
Replication	All the effects that are reported are ones that were replicated across the group of individuals tested; they were found across the whole group of individuals in a mixed effects analysis.
Randomization	The order of conditions (active TUS or sham TUS was randomised across participants.
Blinding	The study was double-blinded, with both researchers and participants blinded to the experimental condition.

Reporting for specific materials, systems and methods

We require information from authors about some types of materials, experimental systems and methods used in many studies. Here, indicate whether each material, system or method listed is relevant to your study. If you are not sure if a list item applies to your research, read the appropriate section before selecting a response.

Materials & experimental systems

n/a	Involved in the study
☒	☐ Antibodies
☒	☐ Eukaryotic cell lines
☒	☐ Palaeontology and archaeology
☒	☐ Animals and other organisms
☒	☐ Clinical data
☒	☐ Dual use research of concern
☒	☐ Plants

Methods

n/a	Involved in the study
☒	☐ ChIP-seq
☒	☐ Flow cytometry
☐	☒ MRI-based neuroimaging

Plants

Seed stocks

Report on the source of all seed stocks or other plant material used. If applicable, state the seed stock centre and catalogue number. If plant specimens were collected from the field, describe the collection location, date and sampling procedures.

Novel plant genotypes

Describe the methods by which all novel plant genotypes were produced. This includes those generated by transgenic approaches, gene editing, chemical/radiation-based mutagenesis and hybridization. For transgenic lines, describe the transformation method, the number of independent lines analyzed and the generation upon which experiments were performed. For gene-edited lines, describe the editor used, the endogenous sequence targeted for editing, the targeting guide RNA sequence (if applicable) and how the editor was applied.

Authentication

Describe any authentication procedures for each seed stock used or novel genotype generated. Describe any experiments used to assess the effect of a mutation and, where applicable, how potential secondary effects (e.g. second site T-DNA insertions, mosiacism, off-target gene editing) were examined.

Magnetic resonance imaging

Experimental design

Design type

Resting-state fMRI BOLD analysis (during either rest or tonic cold pain stimulus)

Design specifications

Each participant underwent three functional MRI scans during each scanning session (baseline, tonic pain and post-pain).

Behavioral performance measures

Participants provided a pain rating at the end of each tonic cold pain stimulus, on a scale between 0 - no pain, and 100 - worst pain imaginable.

Acquisition

Imaging type(s)

Structural and functional

Field strength

3 Tesla

Sequence & imaging parameters

Data were collected on a Siemens MAGNETOM Prisma 3T scanner with a 64-channel head coil. At visit 1, a T1-weighted structural scan and a pointwise encoding time reduction with radial acquisition (PETRA) scan were collected for use in acoustic simulations and neuronavigation. The T1-weighted structural scan was acquired with an MPRAGE sequence with a TR of 2.1s, echo time of 2.26ms, inversion time of 900ms, flip angle of 8°, GRAPPA acceleration factor of 2, matrix size of 256×256, 176 slices, and with 1mm³ isotropic voxels). The PETRA was acquired with TR of 3.61ms, echo time of 0.07ms, flip angle of 8°, 320 slices per slab and slice thickness of 0.75mm. 3D distortion correction was applied following acquisition.

At visits 2 and 3, three resting-state BOLD scans were completed (rest/baseline, tonic cold pain, and post-pain recovery), followed by a field map and T1-weighted structural scan, and finally a magnetic resonance spectroscopy (MRS) scan. Due to an upgrade of the scanner software, the second 14 datasets were collected with a slightly modified scan sequence than the first 15 (for BOLD and MRS scans only). Modified parameters are indicated in italic text following the initial parameter used if applicable. Resting-state BOLD fMRI data were acquired using a multi-echo echo-planar imaging (EPI) sequence with repetition time (TR) of 1.5s / 1.55s, 4 echo times (11.0, 27.25, 43.5, and 59.75ms / 13.6, 29.86, 46.1 and 62.36ms), flip angle of 77°, 2.6mm³ isotropic voxels, 51 axial slices, interleaved acquisition, 220mm field of view, matrix size of 84×84, 2.6mm slice thickness, GRAPPA acceleration factor of 2, multi-band acceleration factor of 3, bandwidth of 2480Hz/Px, and 240 volumes per run. The field map was acquired to enable correction of field inhomogeneity during analysis, with 2.6mm3 isotropic voxels and 220mm field of view. The T1-weighted scan had the same parameters as for visit 1. MRS data was acquired in a 3.5x2.5x2.5cm3 voxel positioned in the dACC (TUS target

region) using a MEGA-PRESS sequence to quantify GABA concentrations, with TR of 2s, echo time of 68ms, excitation flip angle of 90°, and 256 / 128 averages. Water suppression was achieved using a frequency-selective saturation pulse (bandwidth = 35Hz) / VAPOUR (bandwidth = 60Hz), and MEGA editing pulses were centred at 1.9ppm and 7.5ppm to detect GABA, with editing pulse flip angle of 180°. Spectral data were collected with a vector size of 2048, bandwidth of 2000Hz / 1850Hz, and acquisition duration of 1024ms / 1107ms. A water unsuppressed reference was collected with 8 averages. Automatic frequency and shim adjustments were applied prior to acquisition.

Area of acquisition: A whole brain scan was used for structural and functional MRI acquisitions.

Diffusion MRI: ☐ Used ☒ Not used

Preprocessing

Preprocessing software: fMRI preprocessing was performed using tools from the FMRIB Software Library v6.0 (FSL; www.fmrib.ox.ac.uk/fsl). Pre-processing included MCFLIRT motion correction, B0 inhomogeneity correction, brain extraction, spatial smoothing (5 mm FWHM), highpass filtering (0.01 Hz).

Normalization: fMRI data were normalised to the MNI standard space via a linear transform (FSL FLIRT) to each individual's high-resolution T1-weighted MRI and a non-linear transform to the MNI152 template (FSL FNIRT).

Normalization template: MNI152_T1_2mm_brain (as packaged with the FSL software)

Noise and artifact removal: Motion outliers were identified using the fslmotionoutliers tool and were included as nuisance covariates in the model.

Volume censoring: We did not remove volumes during which significant movement occurred, instead, we used our motion-related artifacts (i.e. regression of motion parameters) as regressors of non interest that were not convolved in our general linear models.

Statistical modeling & inference

Model type and settings: Resting state fMRI data were analysed using tools in FMRIB Software Library v6.0 (FSL). Group-level whole brain, mixed effects analysis with a cluster-based correction for multiple comparisons was performed using FEAT to search for differences in functional connectivity with the dACC seed-region and the rest of the brain, between the active TUS and sham TUS conditions. To explore changes in resting state network connectivity, probabilistic independent component analysis (ICA) as implemented in MELODIC was used. Data from both active and sham TUS sessions were pre-processed by masking non-brain voxels, applying voxel-wise demeaning and normalisation of the voxel-wise variance, then projected into a 20-dimensional subspace using principal component analysis. Spatial maps from this group-average analysis were used to generate subject-specific versions, and associated timeseries, using dual regression. Group differences between the active TUS and sham TUS conditions were then compared using randomise with threshold-free cluster enhancement (TFCE; 5000 permutations, p<0.05).

Effect(s) tested: The effect tested was a measure of whole-brain functional connectivity of the seed region of interest derived from the voxel-wise whole-brain general linear model described above.

Specify type of analysis: ☐ Whole brain ☐ ROI-based ☒ Both

Anatomical location(s): The mask for the dACC seed-region was generated by creating 5mm spheres around each of the 3 targets, combining these, and then warping this mask to each individual participant's functional space. For each participant, any regions of the dACC mask that overlapped with thresholded white matter or cerebrospinal fluid masks were subtracted, to create an individualised seed in grey matter only.

Statistic type for inference (See Eklund et al. 2016): Whole-brain Z-statistic maps were thresholded using clusters determined by a one-sided t-test (corresponding to a p = 0.05) and a FDR cluster significance threshold of p = 0.05.

Correction: FDR-corrected cluster significance threshold of p = 0.05

Models & analysis

n/a	Involved in the study
☐	☒ Functional and/or effective connectivity
☒	☐ Graph analysis
☒	☐ Multivariate modeling or predictive analysis

Functional and/or effective connectivity: The parameter estimate derived from the voxel-wise whole-brain general linear model was used as a measure of seed-based functional connectivity.
