# Supplementary material for: Multi-focal ultrasound neuromodulation to the dorsal anterior cingulate cortex disrupts behavioural and neural pain processing
Source: Nat Commun. 2026 May 9;17:6269. doi: 10.1038/s41467-026-72934-3 (PMC13376748; doi:10.1038/s41467-026-72934-3)
Supplement: Supplementary file 3 — Transparent Peer Review file [file 41467_2026_72934_MOESM3_ESM.pdf]

nature portfolio

Peer Review File

Multi-focal ultrasound neuromodulation to the dorsal anterior cingulate cortex disrupts behavioural and neural pain processing

Corresponding Author: Dr Sophie Clarke

This file contains all reviewer reports in order by version, followed by all author rebuttals in order by version.

Version 0:

Reviewer comments:

Reviewer #1

(Remarks to the Author)
The authors investigated the neural mechanisms of transcranial focused ultrasound of the dorsal anterior cingulate for pain in 32 healthy individuals. Strengths include the double-blind design, two neuroimaging methods (MRI and MRS) to identify mechanisms of action, and individualized TUS beam modeling and engagement measures. Weaknesses include insufficient rationale for certain study design decisions (e.g. TUS protocol parameters and 3 targets instead of 1, choice of seed regions for connectivity analyses) and insufficient discussion of this study's findings within the current literature. I believe these weaknesses and specific comments below could be addressed to make this suitable for publication in this journal.
Introduction
1. Since this study's main area of novelty appears to be its investigation of the neural basis of TUS' analgesic effects; the authors should make a stronger case for why this is important. It should be noted that a previous dACC TUS study included EEG (Strohman et al., 2024) for example.
2. Be more specific about directionality of GABA and glutamate and pain perception changes in your hypotheses.
Methods
1. Why 3 different TUS sites in the dACC as opposed to 1? And why were these 3 particular sites chosen? Were they stimulated in the same order in each subject?
2. How were TUS stimulation parameters decided? Why choose the frequency and pulse duration targeting each part of the dACC for only 80 seconds?
3. Provide more information about how skull attenuation was addressed.
4. Confirm that the TUS description conforms to ITRUSST's standardized reporting for TUS.
5. Provide more demographic information for your participants.
6. Was there a reason for using an ICA approach rather than a seed-based approach for the salience network analyses? Any reason why the frontoparietal network was not investigated?
7. Any significant differences in your MRI measures of interest pre versus post scanner software upgrade?
Results
1. Were there significant differences in the cold gelled-water stimulus during the different conditions?
2. Are outliers driving the relationship between pain intensity rating and the cold gel temperature during the sham TUS condition?
Discussion
1. Please expand further on potential reasons previous dACC TUS studies found significant pain rating reductions while the present study did not. How do you think the different study designs may have contributed to different results?
2. The authors note that different pain measures may be more sensitive to dACC TUS changes, although the Strohman et al. (2024) study found analgesic effects using a similar pain intensity outcome measure.
3. How were your TUS stimulation protocols similar/different to other dACC TUS studies?
a. Do you think different TUS parameters could have led to analgesic effects?
4. Are your neuroimaging findings consistent with the wider neuroimaging of pain interventions literature?
5. Elaborate on the clinical implications/translational impacts of this study.
Figures
1. Figure 3b: y-axis labels need to be more specific

Reviewer #2

(Remarks to the Author)
I co-reviewed this manuscript with one of the reviewers who provided the listed reports. This is part of the Nature Communications initiative to facilitate training in peer review and to provide appropriate recognition for Early Career Researchers who co-review manuscripts.

Reviewer #3

(Remarks to the Author)
This paper observes changes in pain perception after transcranial ultrasound stimulation (TUS) of the dACC. Moreover, it studies mechanistic links observing the changes in fMRI/functional connectivity and the relation between metabolic changes (GABA/Glx) and pain rations.

Overall, this study in healthy participants extends existing TUS studies by observing multiple time points and combining fMRI with MRS for a deeper understanding of ultrasound stimulation effects.

Major points:
- It would be good to have a supplementary table that compares this study with the two previous studies. For example, did previous studies also use the 10Hz pattern used here? If not, this might explain some of the differences in the findings.
- active sessions tend to show a larger decrease from T1 to T2 but also a larger increase from T0 to T1. Could this be explained in a framework of neuroplasticity? Say the system is adapting faster from T0 to T1 (becoming more sensitive) but also adapting/habituating faster between T1 and T2 leading to this ‘delayed analgesic’ effect?
- p. 13, line 325: was there any systematic increase in temperature between subsequent time points (e.g. from T0 to T1 or T1 to T2)? Moreover, is there any relationship between the change in pain intensity between T1 and T2 and the change in temperature between T1 and T2?

Minor points:
- Figure 2b: is this over all three timepoints or just T1/T2?
- page 4, line 114: “slight variation in temperature” is an understatement given that sessions could vary by 10 C. Moreover, variation seems larger for the sham sessions.
- Figure 3c legend: please indicate that it is the left DLPFC and cerebellum
- What is the age and sex profile for the 29 (23) participants that were ultimately part of the fMRI (MRS) study?
- p. 12, lines 284-286: scanner software update: I thought this problem was already addressed by excluding 3 participants (line 83)?

Reviewer #4

(Remarks to the Author)
In this study, Clarke and colleagues investigated changes in pain scores and functional measures following TUS of the dACC. They reported a greater reduction in pain ratings between 28 and 55 minutes after active TUS compared to sham. The sample size was relatively small (<30). While this is an important and interesting question for the field, several major concerns limit the strength of the current findings.

1. The main concern is the lack of direct evidence linking the reported effects to TUS of the dACC. For example, no significant differences in pain intensity ratings were observed at any individual time point between active and sham groups. Additionally, there are no differences in GABA concentration between the sham and active TUS groups. Therefore, it is unclear whether the effects described in the manuscript can be attributed to TUS.

2. Since the reported reduction in pain ratings between 28 and 55 minutes in the active group lacks direct supporting evidence, and the statistical significance ($p = 0.049$) is marginal and would not withstand correction for multiple comparisons, the interpretation of the delayed analgesic effect is arbitrary.

3. While the correlation between changes in GABA and pain ratings appears reasonable, there is no evidence that it is attributable to stimulation, given the absence of group differences in GABA between active and sham. Instead, differences in GABA levels may reflect participants’ state at that time, particularly under cold pain exposure.

4. The length of resting-state fMRI (~6 min) is likely insufficient to capture reliable FC changes as suggested by recent work (Ooi et al., 2025).
Reference: Ooi, Leon Qi Rong, et al. "Longer scans boost prediction and cut costs in brain-wide association studies." Nature (2025): 1-10.

5. It may be helpful for the authors to report whether FC differences between the active and sham groups were observed during the first and third runs of rs-fMRI. The first fMRI run is closer to the stimulation and may have a higher likelihood of capturing such FC changes.

6. Without an assessment of blinding effectiveness, it is difficult to determine whether participants remained blinded

throughout the experiment.

Version 1:

Reviewer comments:

Reviewer #1

(Remarks to the Author)
Thank you for your thorough and thoughtful responses. There are a few remaining questions to be addressed:
Introduction
1. The phrasing of the new hypothesis is currently a bit confusing. I would recommend rewriting as "During painful heat stimulation, ACC Glx increases and GABA decreases; therefore, during TUS-induced analgesia (decreased pain intensity), we hypothesized that ACC Glx would decrease and GABA would increase."
Methods
1. The authors responded that the multi-focal approach was used to maximize target engagement since TUS is focally specific, which highlights the importance of the specific target site within the dACC. Can you add dACC target site information/coordinates to the new Supplementary Table 3 for the previous dACC TUS studies? Were there meaningful differences in the dACC sites chosen for this study compared to other dACC TUS studies which found analgesic effects?
2. Since the three dACC TUS sites were always stimulated in the same order in each subject, is there any potential for order effects?
3. Thank you for providing more rationale for the sonication parameters. My question remains about the choice to sonicate for 80 seconds.

Reviewer #2

(Remarks to the Author)
I co-reviewed this manuscript with one of the reviewers who provided the listed reports. This is part of the Nature Communications initiative to facilitate training in peer review and to provide appropriate recognition for Early Career Researchers who co-review manuscripts.

Reviewer #3

(Remarks to the Author)
The authors have successfully addressed all comments.

Reviewer #4

(Remarks to the Author)
I would like to thank the authors for their thorough revision of the manuscript. They have addressed all of my concerns.

Open Access This Peer Review File is licensed under a Creative Commons Attribution 4.0 International License, which permits use, sharing, adaptation, distribution and reproduction in any medium or format, as long as you give appropriate credit to the original author(s) and the source, provide a link to the Creative Commons license, and indicate if changes were made.
In cases where reviewers are anonymous, credit should be given to 'Anonymous Referee' and the source.
The images or other third party material in this Peer Review File are included in the article's Creative Commons license, unless indicated otherwise in a credit line to the material. If material is not included in the article's Creative Commons license and your intended use is not permitted by statutory regulation or exceeds the permitted use, you will need to obtain permission directly from the copyright holder.
To view a copy of this license, visit https://creativecommons.org/licenses/by/4.0/

REVIEWER COMMENTS

<u>Reviewer #1 (Remarks to the Author):</u>

The authors investigated the neural mechanisms of transcranial focused ultrasound of the dorsal anterior cingulate for pain in 32 healthy individuals. Strengths include the double-blind design, two neuroimaging methods (MRI and MRS) to identify mechanisms of action, and individualized TUS beam modeling and engagement measures. Weaknesses include insufficient rationale for certain study design decisions (e.g. TUS protocol parameters and 3 targets instead of 1, choice of seed regions for connectivity analyses) and insufficient discussion of this study's findings within the current literature. I believe these weaknesses and specific comments below could be addressed to make this suitable for publication in this journal.

Thank you for this detailed and very helpful review of the manuscript. Overall, we have addressed this feedback by adding more detailed information about the study design choices and expanded more on the implications of the study's findings in the Discussion section. Below we have addressed each of the specific comments in detail.

Introduction

1. Since this study's main area of novelty appears to be its investigation of the neural basis of TUS' analgesic effects; the authors should make a stronger case for why this is important. It should be noted that a previous dACC TUS study included EEG (Strohman et al., 2024) for example.

Thank you for highlighting this as we agree it is essential that it is clear why this is important. To make a stronger case for this, we have added the following sentences in to the Introduction section: *'Gaining this mechanistic understanding for the neural basis of effects is valuable in progressing the utility of TUS for pain; by identifying the brain regions and circuits involved (supporting optimal target selection) and by understanding whether TUS-induced effects can produce longer-term analgesia through network reorganisation. Crucially, this enhanced understanding of the neural basis of effects also enables a deeper insight to how TUS could be optimally adapted to treat different pain conditions, given the significant heterogeneity in the neural circuits and mechanisms involved in different types of pain.'*

The previous study by Strohman and colleagues applied EEG using only two electrodes to measure the contact-heat evoked potential (CHEP), which is very different to a whole-brain EEG approach that could have looked at large-scale network dynamics across the whole brain. This aspect is what we are particularly interested in with the current study; to understand how TUS applied to one brain region (the dACC) can result in analgesia via network-level effects across the pain processing system. We used fMRI to explore these neural effects in the period after the stimulation, as it also offers additional benefits in spatial specificity and ability to explore neural changes in deep brain regions, enabling very different aspects of the neural basis for TUS effects to be explored with this design compared to the Strohman et al. study. We do already reference that study in the Introduction section in lines 44-45 where we introduce previous studies that have demonstrated effectiveness of both online and offline TUS in modulating pain.

2. Be more specific about directionality of GABA and glutamate and pain perception changes in your hypotheses.

Since TUS is still an emerging neurotechnology in humans, there are only a few studies investigating TUS-induced effects on pain processing, and the complexity of the parameter space is not yet fully explored, it is difficult to make specific hypotheses about the TUS-induced effects. The aim for our study and the selected TUS protocol was that it would be inhibitory, following multiple reports in non-human primates (NHP) (Folloni et al., 2019; Verhagen et al., 2019; Folloni et al., 2021). Therefore, we would hypothesise that it would result in analgesia and behavioural measures of pain perception (i.e. the reported pain ratings) would decrease.

Based on wider literature, there is evidence that experimental heat pain in healthy participants elicits increased Glx and decreased GABA concentration in the ACC compared to rest – *"ascribed to elevated metabolic and neurotransmitter turnovers of Glu due to the increased activity of the excitatory system, while reduced GABA+ levels may reflect the down-regulation of inhibitory neurotransmission during pain processing"* (Cleve et al.2015). Based on this, we would hypothesise that the TUS would result in decreased Glx and increased GABA (associated with a decrease in pain / analgesic effect).

Furthermore, there is evidence that increased GABA levels in the ACC correlate with improved clinical outcomes for patients with migraine, with decreased frequency, intensity and disability over time. (Peek et al. 2021).

In the Introduction section, we have added this more specific hypothesis at the end of the section: *'Specifically, since TUS has been shown to result in analgesia in previous studies, we hypothesised that the behavioural measure of pain perception would show decreased pain ratings. It has been shown that ACC Glx increases significantly during painful heat stimulation while GABA decreases at the same time[19], so by extension we also hypothesised that GABA and Glx concentrations would increase and decrease, respectively, during TUS-induced analgesia resulting in decreased pain intensity.'*

References:

Folloni D, Verhagen L, Mars RB, Fouragnan E, Constans C, Aubry JF, Rushworth MFS, Sallet J. Manipulation of Subcortical and Deep Cortical Activity in the Primate Brain Using Transcranial Focused Ultrasound Stimulation. Neuron. 2019 Mar 20;101(6):1109-1116.e5. doi: 10.1016/j.neuron.2019.01.019.

Verhagen L, Gallea C, Folloni D, Constans C, Jensen DE, Ahnine H, Roumazeilles L, Santin M, Ahmed B, Lehericy S, Klein-Flügge MC, Krug K, Mars RB, Rushworth MF, Pouget P, Aubry JF, Sallet J. Offline impact of transcranial focused ultrasound on cortical activation in primates. Elife. 2019 Feb 12;8:e40541. doi: 10.7554/eLife.40541

Davide Folloni et al., Ultrasound modulation of macaque prefrontal cortex selectively alters credit assignment–related activity and behavior. Sci. Adv.7,eabg7700(2021).DOI:10.1126/sciadv.abg7700

Cleve M, Gussew A and Reichenbach J R. (2015). In vivo detection of acute pain-induced changes of GABA+ and Glx in the human brain by using functional 1H MEGA-PRESS MR spectroscopy. Neuroimage. 105;67-75. https://doi.org/10.1016/j.neuroimage.2014.10.042

Peek AL, Leaver AM, Foster S, Puts NA, Oeltzschner G, Henderson L, Galloway G, Ng K, Refshauge K, Rebbeck T. Increase in ACC GABA+ levels correlate with decrease in migraine frequency, intensity and disability over time. J Headache Pain. 2021 Dec 13;22(1):150. doi: 10.1186/s10194-021-01352-1.

Methods

1. Why 3 different TUS sites in the dACC as opposed to 1? And why were these 3 particular sites chosen? Were they stimulated in the same order in each subject?

The multi-focal approach with three sites in the dACC were used because the dACC, considering anatomical landmarks, is a much larger region than the size of the focal TUS beam, so in order to maximise target engagement and produce a neuromodulatory effect across the dACC we opted to use the 3 stimulation sites. This also resulted in 3 x the amount of TUS applied overall, spread across the cortical region. These 3 particular sites were chosen based on the Harvard-Oxford cortical atlas mask for the anterior cingulate cortex, with the sites located in the dorsal portion of the ACC region (see image below). The three sites were always stimulated in the same order in each subject, always starting with target A, then B, then C.

Additionally, this is beneficial for assessing the TUS-induced effects using MRS, since the MRS voxel cannot be too small (due to signal to noise constraints), so applying three TUS sonications covers a larger area of the MRS voxel overall.

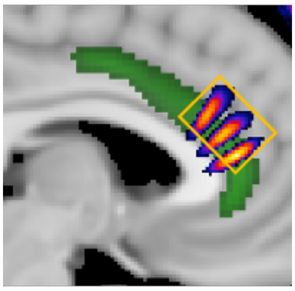

Figure: Harvard-Oxford Atlas mask for anterior cingulate cortex, with stimulated sites overlaid (within the dorsal ACC) and example MRS voxel in yellow.

Changes have been made in the Methods section '4.4 TUS stimulation protocol' to address this comment, including the following sentences: '*A multi-site approach, with 3 sites within the dACC, was used in order to maximise target engagement and subsequently the neuromodulatory effect, since the dACC is a relatively large region compared to the focal size of the TUS beam.*' And '*…in the same order from A to C in all participants.*'

2. How were TUS stimulation parameters decided? Why choose the frequency and pulse duration targeting each part of the dACC for only 80 seconds?

The aim when deciding the TUS parameters was for the sonication to be inhibitory, to reduce activation of the dACC and therefore inhibit the dACC's role in encoding affective aspects including the salience or unpleasantness of pain signals. The specific TUS parameters were decided based on a number of factors. For the pulse repetition frequency (PRF), we used 10Hz which is aligned to alpha oscillations associated with inhibition in the brain (with the same rationale as using 5Hz patterned protocol). There are many studies showing evidence of inhibitory or suppressive effects of a 10Hz PRF in NHPs (Folloni et al., 2019; Verhagen et al., 2019; Folloni et al., 2021). In addition, we were also inspired by previous work by Badran et al. 2020, who showed altered pain responses with this PRF, and we also had collected some pilot data in our lab (although with a different pain task), which indicated the 10Hz protocol produced an analgesic effect. In addition, to maximise neuromodulatory effects, we selected a 10% duty cycle to maximise pulse duration and therefore radiation force while keeping the thermal

rise as low as possible. We were careful to remain under the non-significant mechanical and thermal risk levels highlighted by ITRUSST.

The following text has been added to the Methods section '4.4 TUS stimulation protocol' to address this comment and provide further information on the choice of sonication parameters: '*We opted for this 10Hz, 10% duty-cycle protocol to produce inhibitory neuromodulation of the dACC, based on its alignment with alpha-frequency inhibitory dynamics and prior evidence of analgesic effects*[6,41-43].'

References:

Folloni D, Verhagen L, Mars RB, Fouragnan E, Constans C, Aubry JF, Rushworth MFS, Sallet J. Manipulation of Subcortical and Deep Cortical Activity in the Primate Brain Using Transcranial Focused Ultrasound Stimulation. Neuron. 2019 Mar 20;101(6):1109-1116.e5. doi: 10.1016/j.neuron.2019.01.019.

Verhagen L, Gallea C, Folloni D, Constans C, Jensen DE, Ahnine H, Roumazeilles L, Santin M, Ahmed B, Lehericy S, Klein-Flügge MC, Krug K, Mars RB, Rushworth MF, Pouget P, Aubry JF, Sallet J. Offline impact of transcranial focused ultrasound on cortical activation in primates. Elife. 2019 Feb 12;8:e40541. doi: 10.7554/eLife.40541

Davide Folloni et al., Ultrasound modulation of macaque prefrontal cortex selectively alters credit assignment–related activity and behavior. Sci. Adv.7,eabg7700(2021).DOI:10.1126/sciadv.abg7700

3. Provide more information about how skull attenuation was addressed.

We agree this is an important aspect to ensure information is adequately provided in the manuscript. We used a four element array with no phase correction. However, we measured our acoustic output in a hydrophone tank (in water) and performed acoustic and thermal simulations through the individual participant's skull given some assumptions on acoustic properties of the different tissues that the ultrasonic beams pass through. These acoustic properties were derived from Hounsfield units obtained through a pseudo-CT generated via a linear mapping from a PETRA (MR image). For each participant we therefore collected a PETRA scan which we converted into a pseudo-CT (Matlab script) to use in k-Plan simulations for each participant. We fixed our ISPPA in free field given conservative derating (see Attali et al., 2023) but additionally performed simulations to compute more realistic losses.

The following statement has been added to the Methods section to provide further information on this: '*The ISPPA in water was maintained at 54W/cm² across participants; with this higher input ISPPA used to account for skull attenuation, which was modelled in the k-Plan acoustic simulations, to ensure safety and to assess the ISPPA reaching the brain target.*'

References

Attali D, Tiennot T, Schafer M, Fouragnan E, Sallet J, Caskey CF, Chen R, Darmani G, Bubrick EJ, Butler C, Stagg CJ, Klein-Flügge M, Verhagen L, Yoo SS, Pauly KB, Aubry JF. Three-layer model with absorption for conservative estimation of the maximum acoustic transmission coefficient through the human skull for transcranial ultrasound stimulation. Brain Stimul. 2023 Jan-Feb;16(1):48-55. doi: 10.1016/j.brs.2022.12.005. Epub 2022 Dec 19. PMID: 36549480.

4. Confirm that the TUS description conforms to ITRUSST's standardized reporting for TUS.

This has been carefully checked and the majority of information required for ITRUSST's standardized reporting of TUS was already included in the manuscript. To ensure this is easily followed by future readers, a new table has been added to the Supplementary Information that includes the completed checklist from the standardised reporting consensus (Martin et al. 2024). One area that was previously omitted from our manuscript was the free-field acoustic parameters, and these have now been added within that new supplementary table: 'Supplementary Table 2: ITRUSST consensus on standardised reporting for transcranial ultrasound stimulation checklist'. This is referred to in the revised Manuscript in the Methods section '4.4 TUS stimulation protocol'.

5. Provide more demographic information for your participants.

We have added a further breakdown of age and sex for each sub-population of participants included in each analysis (fMRI and MRS) as follows:

For the 29 participants included in the fMRI analyses, the mean age was 25.6±9.5 years, range 21-66, 19 female, 10 male (sex and gender aligned by self-report). This has been added in line 90-91 at the first inclusion of the fMRI sample size.

For the 23 participants included in the MRS analyses, the mean age was 26.6±11.3 years, range 21-66, 14 female, 9 male (sex and gender aligned by self-report). This has been added in line 469-470 with the data cleaning information that outlines the MRS sample size.

Further demographic data, such as ethnicity or level of education, was not collected and therefore cannot be added into the manuscript.

6. Was there a reason for using an ICA approach rather than a seed-based approach for the salience network analyses? Any reason why the frontoparietal network was not investigated?

While seed-based approaches are excellent for node-to-node connectivity to explore specific relationships between the seed and other regions of the brain, as we have applied them in the study, there are many additional advantages of applying an ICA-based approach for network analyses that we wanted to capitalise on. The ICA approach is data-driven, requiring no a priori seed and therefore avoiding any bias from the specific seed used. It is able to decompose signals that share anatomical space, which is ideal for isolating the salience network from others that may overlap (such as the executive control network or the default mode network, which may both have overlap with the dACC at certain times). ICA also offers benefits in isolating and removing noise components such as motion and physiological noise, potentially improving signal quality.

The following sentence has been added to the methods to highlight these benefits: *"These two approaches offer different strengths, with seed-based analysis enabling exploration of targeted hypotheses about connectivity between the dACC seed and other brain regions, whereas the ICA-based analysis enabled data-driven, whole-network exploration without prior assumptions such as a predefined seed-region."*

Regarding the frontoparietal network, this was investigated, since the ICA-based approach identifies a set number of components (in our case 20), corresponding to spatially independent patterns of brain activity, hence the approach can identify multiple networks simultaneously without prior assumptions. The frontoparietal network was identified within these components. Due to the fact that the dACC is a core node of the salience network, this was of particular interest, so we identified the component

that corresponded with this network and focussed on this when presenting the results. The salience network component was the only one that shown a significant difference between the active TUS and sham TUS conditions.

7. Any significant differences in your MRI measures of interest pre versus post scanner software upgrade?

We have conducted a two-way mixed ANOVA on the BOLD parameter estimates for the two regions that showed a significant difference in the whole-brain seed-based analyses (the SMA and pre-motor cortex). For the SMA, this confirmed that there was a significant main effect of condition (active/sham; $F(1,27) = 14.40$, $p = 0.0008$), but that there was no main effect of scanner update (pre/post, $F(1,27) = 0.01$, $p = 0.92$) and no significant condition × update interaction ($F(1,27) = 0.20$, $p = 0.66$), suggesting that the scanner update did not influence the condition effects.

Similarly for the pre-motor cortex, this confirmed that there was a significant main effect of condition (active/sham; $F(1,27) = 7.5$, $p = 0.01$), but that there was no main effect of scanner update (pre/post, $F(1,27) = 0.005$, $p = 0.95$) and no significant condition × update interaction ($F(1,27) = 0.67$, $p = 0.42$), suggesting that the scanner update did not influence the condition effects.

It is important to note that the group sizes for pre- and post-scanner update are n=15 and n=14 respectively, so this analysis will be underpowered given the small group sizes.

The plots below illustrate the data pre- and post- scanner upgrade, showing there are not large discrepancies between the datasets.

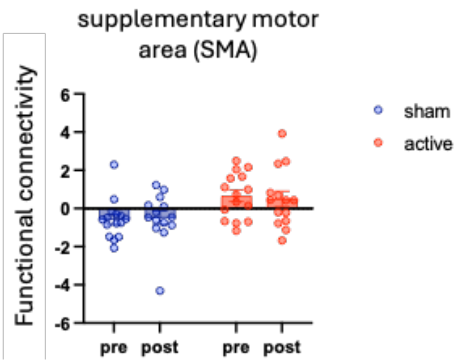

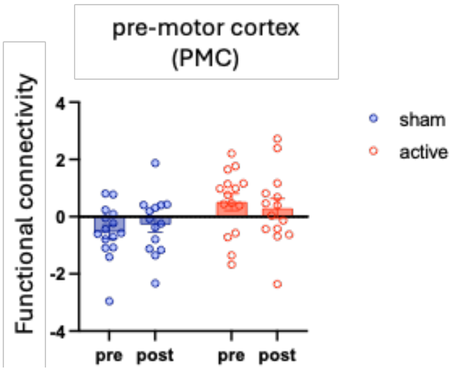

Results

1. Were there significant differences in the cold gelled-water stimulus during the different conditions?

There were no significant differences between the temperature of the gel stimulus between the active and sham conditions for either all three time points pooled data (paired t-test, p=0.59), or for any of the time points individually (T0; p=0.76, T1; p=0.76, T2; p=0.75).

The following plots have been added to the Supplementary Information in 'Supplementary Figure 2: Temperature of cold gel stimulus for all time points pooled (far left) and for T0, T1 and T2 time points separately', and are referenced in the Results section '2.2 Behavioural results' as follows: '*Control analyses were completed to ensure there were no significant differences in temperature between the active and sham conditions that may influence the results. This confirmed that there were no significant*

differences between the temperature of the gel stimulus between the active and sham conditions for either all three time points pooled data (paired t-test, p=0.59), or for any of the time points individually (T0; p=0.76, T1; p=0.76, T2; p=0.75). Plots are shown in Supplementary Figure 2.'

Supplementary Figure 2: Temperature of cold gel stimulus for all time points pooled (far left) and for T0, T1 and T2 time points separately

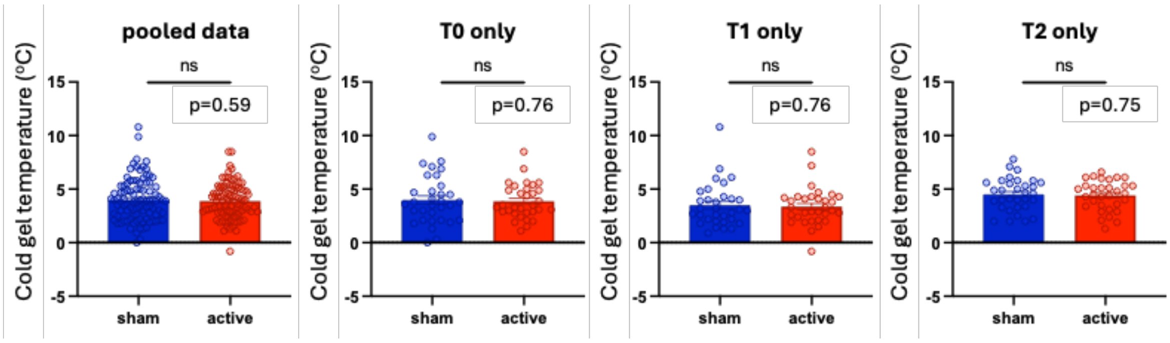

2. Are outliers driving the relationship between pain intensity rating and the cold gel temperature during the sham TUS condition?

To assess whether the linear relationship between pain intensity rating and the cold gel temperature in the sham condition was driven by outliers, we have repeated the analysis using robust regression (*robustfit* in MATLAB), which down-weights data points with disproportionately large residuals. The robust model produced a slope of –4.04 (t = –3.15, p = 0.002), very similar to the simple linear regression result in the original manuscript (p = 0.003). The robust weights showed that only a small number of observations received reduced influence, indicating that a few moderately extreme points added noise but did not drive the effect. Importantly, the regression remained significant after down-weighting these observations, demonstrating that the relationship reflects a stable underlying effect rather than being attributable to outliers. For completeness we have repeated this additional analysis for the active TUS condition. (which also confirms the original result was not attributable to outliers).

This has been added to the Results section '2.2 Behavioural results' as follows: *'Robust linear regression confirmed the patterns observed. In the sham condition, temperature was negatively associated with rating (slope = –4.04, t(94) = –3.15, p = 0.002), consistent with the original result. In the active condition, there was no association (slope = –0.29, t(94) = –0.29, p = 0.78), and in both cases a few moderately extreme points were down-weighted without altering the overall conclusion, confirming that the observed effects (or lack thereof) were not driven by outliers.'* A note has also been added to the Methods section '4.7 Statistical analysis' in relation to this additional analysis.

Discussion

1. Please expand further on potential reasons previous dACC TUS studies found significant pain rating reductions while the present study did not. How do you think the different study designs may have contributed to different results?

We have expanded on the potential reasons for the differences between the findings of Strohman et al. and Riis et al. showing significant pain rating reductions and our study in the Discussion section.

Following the statement about the pain intensity rating results not being replicated, the following has been added: *"This could be due to differences in the type of pain being assessed or differences in the TUS intervention delivered. Tonic stimuli, such as the cold pain applied here, are more temporally complex and have been shown to be differentially encoded in the brain compared to acute/phasic stimuli, relying more on emotional and evaluative circuits[23]. In addition, although experimental tonic pain has more similar network-level representations to clinical pain[24], the fact that TUS was delivered for much longer and with different parameters may have resulted in the differential effects on pain intensity ratings between our results and that of Riis et al."*

2. The authors note that different pain measures may be more sensitive to dACC TUS changes, although the Strohman et al. (2024) study found analgesic effects using a similar pain intensity outcome measure.

The differences with the Strohman et al. study are outlined in the changes made for the previous comment, and although they did see changes in pain intensity ratings in that study, we still think that the addition of the further measures on pain unpleasantness and mood would have been interesting and could have been more sensitive to TUS-induced changes to the dACC's specific role in pain processing in our study.

3. How were your TUS stimulation protocols similar/different to other dACC TUS studies? Do you think different TUS parameters could have led to analgesic effects?

Related to this comment, a table comparing the stimulation protocols used in other, recent pain TUS studies was requested in a comment from Reviewer #3 (see below), and has been added to the Supplementary Materials in Supplementary Table 3, and referenced in the manuscript in the Methods section as follows: "*Additionally, a table including the stimulation parameters from three other pain TUS studies in included in Supplementary Table 3 to enable comparison of protocols used*."

It is reproduced below:

Supplementary Table 3: TUS protocols for recent pain studies

Study	Device	Design and brain target	Ff (kHz)	PRF (Hz)	Pulse duration (ms)	Duty cycle (%)	Sonication duration	Pulse repetition interval	Number of sonications	Average peak pressure (MPa)
Badran et al. 2020	BrainSonix BXPulsar 1002	Offline, targeting thalamus	650	10	5	5%	30s	30s	10	0.72
Strohman et al. 2024	Sonic Concepts, model H-104	Online, targeting dACC	500	1000	0.36	36%	100ms	1ms	40	0.1152

Riis et al. 2024	Bespoke device with 2 phased array Transducers (see paper for details)	Offline, targeting dACC	650	100<br><br>(Pulse Train RF: 1.42)	5<br><br>(Train duration: 30)	50%<br><br>(30%)	30ms<br><br>(3 minutes)	10ms<br><br>(1000ms, off period = 700ms)	12 (across 4 targets)	1
**Current study**	**NeuroFUS CTX-500-4**	**Offline, targeting dACC**	**500**	**10**	**10**	**10%**	**80s**	**90ms**	**3 (multi-focal)**	**0.354**

This table shows that there were similarities and differences with the TUS stimulation protocols used in other dACC TUS studies. The protocol used by Strohman et al. (2024) was the most different, given this was an online study investigating real-time effects of the TUS on pain responses. The protocol used by Badran et al. (2020) used the same pulse repetition frequency (PRF) of 10Hz, with similar pulse duration and duty cycle. The protocol used by Riis et al. (2024) was quite different again, with a lower PRF of 1.42Hz and higher duty cycle of 50%.

The optimisation of TUS parameters for eliciting maximal TUS effects is an area of ongoing research, and there are a number of challenges faced, such as the vast parameter space and the large number of applications to different brain regions and different functions. Different TUS parameters are very likely to produce different effects, in fact even the same TUS parameters may elicit different effects in different brain regions, since effects are likely to depend on a number of factors such as the relative proportion of inhibitory versus excitatory neurons, or concentrations of different neurotransmitters. A recent review by Nandi et al. (2024) outlines the current status of evidence across in vitro, animal and human studies, aiming to support evidence-based parameter selection, although there are several important gaps remaining on the optimal frequencies for pulsed protocols and optimal duty cycles.

The following has been added to the Discussion section – linking to the differences in the findings from other dACC TUS studies: *"More broadly, the TUS parameters and brain targets used across pain studies vary, and the optimisation of TUS parameters for eliciting maximal TUS effects is an area of ongoing research. It is likely that different stimulation parameters could have resulted in stronger analgesic effects. Ongoing work in the field aims to support evidence-based parameter selection, although gaps remain in identifying optimal frequencies for pulsed protocols and optimal duty cycles[25]."*

4. Are your neuroimaging findings consistent with the wider neuroimaging of pain interventions literature?

This is an interesting aspect on which we agree further discussion would improve the manuscript. There are a number of emerging neuromodulation techniques that are able to selectively target specific brain regions for pain modulation, and we have focussed on these since they are comparable to the TUS technique targeting the dACC used in this study. Particularly there are recent studies highlighting motor-related regions as promising targets for neuromodulation, linking to the finding in this study that connectivity was altered between the dACC and regions related to action planning (including the pre-motor cortex and SMA).

The following section has been added to the Discussion section of the manuscript in relation to this: *'The neuroimaging results from this study show altered connectivity between the dACC and motor-related regions highly connected to the primary motor cortex (the pre-motor cortex and supplementary*

motor area). Recent neuroimaging research has identified the somato-cognitive action network (SCAN); a set of inter-effector regions within the primary motor cortex shown to be activated during action planning and highly connected to the salience network (which includes the dACC and is key for pain processing). Personalised and precision targeting of the SCAN and salience network using direct cortical stimulation (DCS) has been shown to be effective in reducing pain for patients with intractable chronic pain[23,24], and repetitive transcranial magnetic stimulation (rTMS) targeting the motor cortex reduced pain intensity in patients with neuropathic pain[25]. In this context, our neuroimaging findings contribute to the growing body of evidence for the application of targeted neuromodulation techniques to regions associated with the SCAN and salience networks.'

Elaborate on the clinical implications/translational impacts of this study.

The mechanistic insights provided by the neuroimaging measures included in the study design provide novel and valuable information that does have clinical implications and translational impacts, particularly focussed on optimising how TUS can be applied to treat chronic pain. To add more information and elaborate on this in the manuscript, we have added the following into the second to last paragraph of the Discussion section: *"By demonstrating this network-level modulation in regions implicated in chronic pain, this work provides a crucial mechanistic bridge supporting the translation of dACC-TUS into clinical trials for persistent pain conditions. Characterising the specific changes in dACC-centred network dynamics highlights potential biomarkers and therapeutic targets that could guide target optimisation and patient selection in future translational studies of TUS for chronic pain."*

Figures

1. Figure 3b: y-axis labels need to be more specific

The y-axis label has been updated to 'Functional connectivity (parameter estimate sampled from the GLM)' – more specific and related to how these plots were generated, to aide interpretation. This has also been updated in the figure legend for 3B and 3C as follows:

***b** Illustrative plots showing the altered connectivity with dACC seed region for the supplementary motor area (left), the pre-motor cortex (right). Plots show the functional connectivity (parameter estimate from the GLM) sampled from each region, for the sham and active conditions.*

***c** Illustrative radar plot showing dACC functional connectivity (parameter estimate from the GLM) sampled from brain regions involved in pain processing, including the two regions that showed significantly altered connectivity in the whole-brain analysis, plus the bilateral dorsolateral prefrontal cortex, thalamus, anterior/posterior insular cortices and the periaqueductal grey and precuneus.*

Reviewer #2 (Remarks to the Author):

I co-reviewed this manuscript with one of the reviewers who provided the listed reports. This is part of the Nature Communications initiative to facilitate training in peer review and to provide appropriate recognition for Early Career Researchers who co-review manuscripts.

<u>Reviewer #3 (Remarks to the Author):</u>

This paper observes changes in pain perception after transcranial ultrasound stimulation (TUS) of the dACC. Moreover, it studies mechanistic links observing the changes in fMRI/functional connectivity and the relation between metabolic changes (GABA/Glx) and pain rations.

Overall, this study in healthy participants extends existing TUS studies by observing multiple time points and combining fMRI with MRS for a deeper understanding of ultrasound stimulation effects.

Thank you for your constructive summary of our study. We appreciate your recognition of the efforts to examine pain perception changes across multiple time points, and to integrate both fMRI-based functional connectivity and MRS-derived metabolic measures. Our goal was to provide a more comprehensive mechanistic perspective on how ultrasound stimulation influences neural and perceptual processes, and we are glad to hear that the combined approach in healthy participants is viewed as a valuable extension of existing TUS research.

Major points:

- It would be good to have a supplementary table that compares this study with the two previous studies. For example, did previous studies also use the 10Hz pattern used here? If not, this might explain some of the differences in the findings.

The table comparing the stimulation protocols used in other, recent pain TUS studies has been added to the Supplementary Materials in Supplementary Table 3, and referenced in the manuscript in the Methods section as follows: “*Additionally, a table including the stimulation parameters from three other pain TUS studies in included in Supplementary Table 3 to enable comparison of protocols used.*”

It is reproduced below:

Supplementary Table 3: TUS protocols for recent pain studies

Study	Device	Design and brain target	Ff (kHz)	PRF (Hz)	Pulse duration (ms)	Duty cycle (%)	Sonication duration	Pulse repetition interval	Number of sonications	Average peak pressure (MPa)
Badran et al. 2020	BrainSonix BXPulsar 1002	Offline, targeting thalamus	650	10	5	5%	30s	30s	10	0.72
Strohman et al. 2024	Sonic Concepts, model H-104	Online, targeting dACC	500	1000	0.36	36%	100ms	1ms	40	0.1152
Riis et al. 2024	Bespoke device with 2 phased array Transducers (see paper for details)	Offline, targeting dACC	650	100<br><br>(Pulse Train RF: 1.42)	5<br><br>(Train duration: 30)	50%<br><br>(30%)	30ms<br><br>(3 minutes)	10ms<br><br>(1000ms, off period = 700ms)	12 (across 4 targets)	1

**Current study**	**NeuroFUS CTX-500-4**	**Offline, targeting dACC**	**500**	**10**	**10**	**10%**	**80s**	**90ms**	**3 (multi-focal)**	**0.354**

Additional discussion has been added to the Discussion section regarding the differences in the stimulation protocols used and the differences in the findings. This is previously outlined in the response to the comments from Reviewer #1 in the Discussion section (please see above).

- Active sessions tend to show a larger decrease from T1 to T2 but also a larger increase from T0 to T1. Could this be explained in a framework of neuroplasticity? Say the system is adapting faster from T0 to T1 (becoming more sensitive) but also adapting/habituating faster between T1 and T2 leading to this 'delayed analgesic' effect?

Yes, we agree that the fact that we observe these larger changes in the pain intensity ratings in the active condition compared to sham could reflect underlying changes in neuroplasticity and responsiveness of the dACC and the wider pain network at each of the time points. It is difficult to make conclusions based on these pain ratings since differences between the active and sham condition overall are not large enough to reach statistical significance, but the observed larger increase in pain intensity between T0 and T1 and the larger decrease between T1 and T2 (illustrated in the plots below) certainly seem to reflect TUS-induced changes in pain sensitivity over time in the active condition, whereas the sham condition remains far more stable.

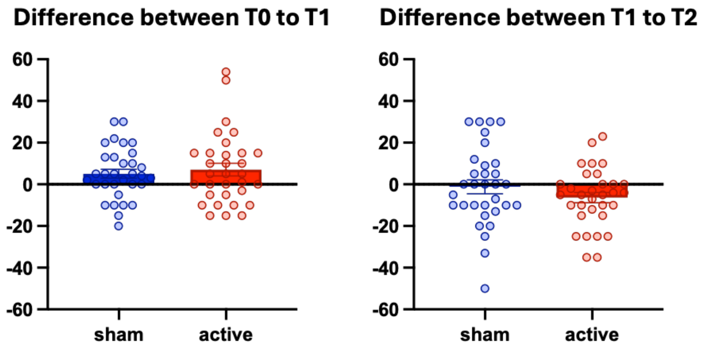

The following has been added to the Discussion section to address this comment: *'There were greater shifts in pain intensity ratings over time in the active TUS condition, with a greater increase between T0 and T1 (although not significantly different) and greater decrease between T1 and T2, compared to sham. This could be explained in a framework of neuroplasticity, potentially reflecting TUS-induced sensitisation at the start shifting to adaptation and pain inhibition over time. Future work is needed to explore these temporal dynamics of TUS effects further.'*

- p. 13, line 325: was there any systematic increase in temperature between subsequent time points (e.g. from T0 to T1 or T1 to T2)? Moreover, is there any relationship between the change in pain intensity between T1 and T2 and the change in temperature between T1 and T2?

This is an important point, and we have conducted some control analyses to assess whether the effects we see with the pain ratings were being driven by differences in the temperature of the cold gel

stimulus. Importantly, as outlined in response to a comment from Reviewer #1 (see above), there were no significant differences between the temperature of the gel stimulus between the active and sham conditions for either all three time points pooled data (paired t-test, p=0.59), or for any of the time points individually (T0; p=0.76, T1; p=0.76, T2; p=0.75). Therefore, we are confident that differences in the pain ratings between the sham and active conditions are not due to changes in the temperature of the stimulus between the two conditions.

The following plots have been added to the Supplementary Information in ‘Supplementary Figure 2: Temperature of cold gel stimulus for all time points pooled (far left) and for T0, T1 and T2 time points separately’, and are referenced in the Results section ‘2.2 Behavioural results’ as follows: ‘*Control analyses were completed to ensure there were no significant differences in temperature between the active and sham conditions that may influence the results. This confirmed that there were no significant differences between the temperature of the gel stimulus between the active and sham conditions for either all three time points pooled data (paired t-test, p=0.59), or for any of the time points individually (T0; p=0.76, T1; p=0.76, T2; p=0.75). Plots are shown in Supplementary Figure 2.*’

Supplementary Figure 2: Temperature of cold gel stimulus for all time points pooled (far left) and for T0, T1 and T2 time points separately

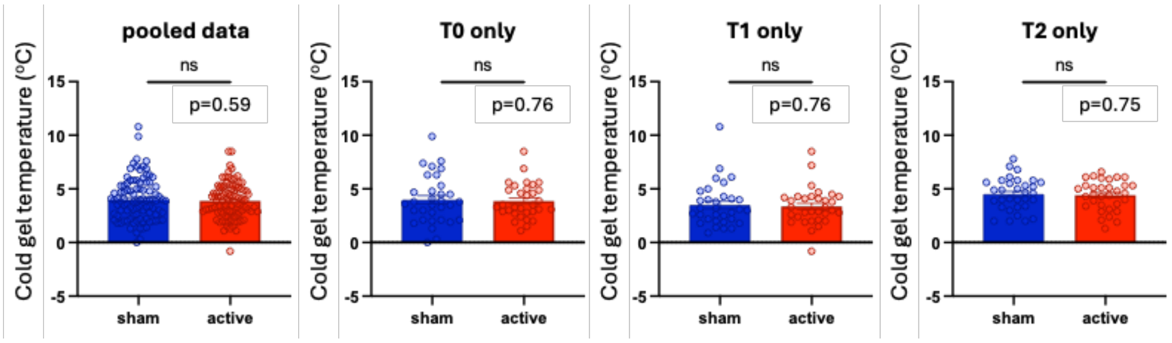

Relating to this specific comment, we have also explored whether there was a systemic increase in temperature between subsequent time points. For gel temperature and time point alone (regardless of active/sham condition), there were significant differences in temperature between time points, as outlined in the plot shown below (asterisks show results from one-way ANOVA with multiple comparisons between each time point). These differences do not show an increase over time, but rather that temperature was lower for T1 than T0, and then higher for T2. This likely reflects logistical challenges with maintaining the temperature of the gels during the experimental session, as at different time points the handling of the gel was impacted by different conditions. For example, at T0 and T1 the gel was used straight away on removal from the fridge, whereas for T2 we had to bring the gel to the MRI control room prior to set-up for the MRS scan, which may account for the slight increase in temperature.

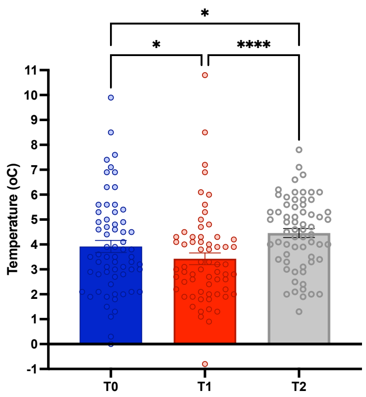

To explore whether the relationship between the change in pain intensity between T1 and T2 and the change in temperature between T1 and T2, we have plotted this for each condition (active and sham). In the sham condition, there is a significant relationship showing that the larger the increase in temperature between T1 and T2, the larger the decrease in pain intensity rating. This relationship is to be expected. Interestingly, this relationship is not present in the active condition. This is concurrent with our previous finding that the expected relationship between pain intensity rating and temperature (that is, lower temperatures correspond to higher pain intensity ratings) is present in the sham conditions but no longer present in the active condition, outlined in Figure 2B in the manuscript.

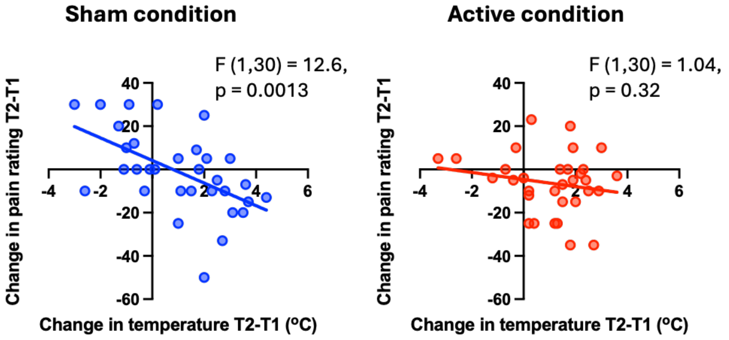

Overall, these analyses indicate that the pain intensity rating results are not being driven by systemic temperature changes of the stimulus over time, particularly since there are no differences in the stimulus temperature between the active and sham conditions. This has been added to the manuscript as outlined above to address this.

Minor points:

- Figure 2b: is this over all three timepoints or just T1/T2?

This is over all three time points. The figure legend for Figure 2B has been updated to clarify this as follows:

b *Relationship between gel temperature and pain intensity ratings pooled for all three time points and plotted for both sham TUS (left) and active TUS (right), showing the expected relationship between temperature and pain intensity is no longer present in the active TUS session.*

- page 4, line 114: “slight variation in temperature” is an understatement given that sessions could vary by 10 C. Moreover, variation seems larger for the sham sessions.

This is a good point, and the word ‘slight’ has been removed to address this. To address the second part of this comment, that the variation seems larger for the sham sessions, please find below the computed descriptive statistics for the temperature of the stimulus by condition. This shows the variation was marginally larger for the sham condition, with range 10.8 compared to 9.3 for the active condition. The mean and standard deviation of the temperature data for each condition is similar. The full outline of descriptive statistics is shown in the table below, as well as a box plot showing the temperature distribution for the active and sham conditions.

	sham	active
Number of values	96	96
Minimum	0.000	-0.8000
Maximum	10.80	8.500
Range	10.80	9.300
Mean	3.993	3.876
Std. Deviation	1.979	1.629
Std. Error of Mean	0.2020	0.1663

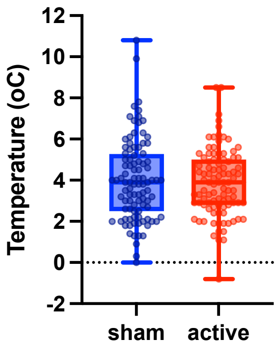

- Figure 3c legend: please indicate that it is the left DLPFC and cerebellum

The figure legend has been updated to indicate it is the left DLPFC and the right cerebellum as follows:

d *Salience network was identified using independent component analysis (ICA) carried out on the full dataset (shown in dotted inset), and subsequent group analysis comparing subject-specific network maps for active TUS and sham conditions showed altered salience network connectivity with the left DLPFC, right cerebellum and right hippocampus.*

- What is the age and sex profile for the 29 (23) participants that were ultimately part of the fMRI (MRS) study?

For the 29 participants included in the fMRI analyses, the mean age was 25.6±9.5 years, range 21-66, 19 female, 10 male (sex and gender aligned by self-report). This has been added in line 90-91 at the first inclusion of the fMRI sample size.

For the 23 participants included in the MRS analyses, the mean age was 26.6±11.3 years, range 21-66, 14 female, 9 male (sex and gender aligned by self-report). This has been added in line 469-470 with the data cleaning information that outlines the MRS sample size.

- p. 12, lines 284-286: scanner software update: I thought this problem was already addressed by excluding 3 participants (line 83)?

There were 3 participants excluded due to a specific image artefact that was introduced as a result of the scanner software update – this specific artefact was then resolved for the remaining datasets. However, remaining datasets were still affected by the upgrade, as the sequence parameters had to

be updated. The specific MRI acquisition parameters used re- and post- scanner upgrade are outlined in the Methods section ‘4.5 Imaging data acquisition (MRI and MRS)’, with the implications discussed in the part of the Discussion outlined in the comment.

Reviewer #4 (Remarks to the Author):

In this study, Clarke and colleagues investigated changes in pain scores and functional measures following TUS of the dACC. They reported a greater reduction in pain ratings between 28 and 55 minutes after active TUS compared to sham. The sample size was relatively small (<30). While this is an important and interesting question for the field, several major concerns limit the strength of the current findings.

We thank the reviewer for the thoughtful and encouraging evaluation of our study. We appreciate the recognition of the importance and interest of the work, and we are grateful for the constructive suggestions. Below we address each point in detail.

1. The main concern is the lack of direct evidence linking the reported effects to TUS of the dACC. For example, no significant differences in pain intensity ratings were observed at any individual time point between active and sham groups. Additionally, there are no differences in GABA concentration between the sham and active TUS groups. Therefore, it is unclear whether the effects described in the manuscript can be attributed to TUS.

We thank the reviewer for this comment, which prompted a deeper examination of the specificity and causal link between TUS and the reported neural effects and whether our fMRI changes could be attributed to TUS or not. We fully acknowledge that the absence of significant differences in pain intensity ratings or GABA concentration between the active and sham conditions limits the direct behavioural and neurochemical evidence for TUS effects. However, our aim was to determine whether TUS nonetheless modulated large-scale network dynamics involving pain and their interaction with the dACC, which may still occur in the absence of overt behavioural or metabolic differences.

To this end, we focused on whether the observed changes in the connectivity fingerprint of the dACC during pain could be uniquely attributed to TUS application, rather than reflecting general session-related variability or nonspecific effects of stimulation.

Control analysis 1

To assess whether the observed modulation of the dACC connectivity fingerprint during pain was specific to the stimulated region and not driven by general session effects, we identified brain regions showing the weakest functional connectivity with the dACC under the SHAM condition. For each of these regions, we computed connectivity fingerprints separately for the TUS and SHAM sessions by constructing matrices of covariation between voxels within the region and an a priori defined constellation of regions used in the main pain analyses (Figure 3C). Pairwise distances between all elements of these matrices were then calculated using the cityblock (Manhattan) distance metric, providing a quantitative measure of dissimilarity between session-specific connectivity profiles. This analysis was designed to identify whether session-related changes in connectivity were confined to the dACC - the site of sonication - or whether similar changes could be observed in regions that were

not directly targeted by TUS. The expectation, if the effects are specific to TUS, is that marked differences should be evident primarily in the dACC and not in these control regions.

The analysis revealed that the dissimilarity in connectivity fingerprints between the SHAM and TUS sessions was significantly greater in the dACC compared with other regions that were not directly sonicated (LG, WM, pINS; new Figure 4A). Specifically, pairwise comparisons showed higher session-related dissimilarity in the dACC relative to all control regions (LG vs dACC: $p = 0.0123$, $t = -2.68$; WM vs dACC: $p = 0.0023$, $t = 3.36$; pINS vs dACC: $p = 3.75 \times 10^{-4}$, $t = 4.06$). These results indicate that the observed modulation of the connectivity fingerprint is localized to the dACC, the site of sonication, and is not present in regions outside the TUS focus.

Taken together, these findings demonstrate that the effects on connectivity fingerprints cannot be attributed to general session differences or a global influence of ultrasound on the entire brain. Instead, they provide evidence that the changes in connectivity patterns are spatially specific to the dACC and therefore most parsimoniously explained by local TUS effects.

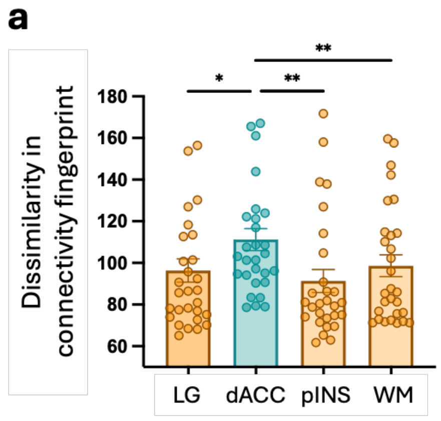

Control analysis 2

We also conducted a seed-based analysis using an alternate seed-region to further determine if observed changes in dACC functional connectivity were related to the TUS stimulation or simply session effects. We selected the lingual gyrus (LG) as the seed-region, since it is not close to the dACC, and created a mask of the same size as the dACC seed mask used, located in grey matter. As shown in new Figure 4B, the functional connectivity between the LG seed region and the same ROIs included in previous analyses was not altered between the active and sham TUS conditions. This adds further evidence that the changes we do see with the dACC seed are not just due to session effects, but an actual effect of the TUS applied there.

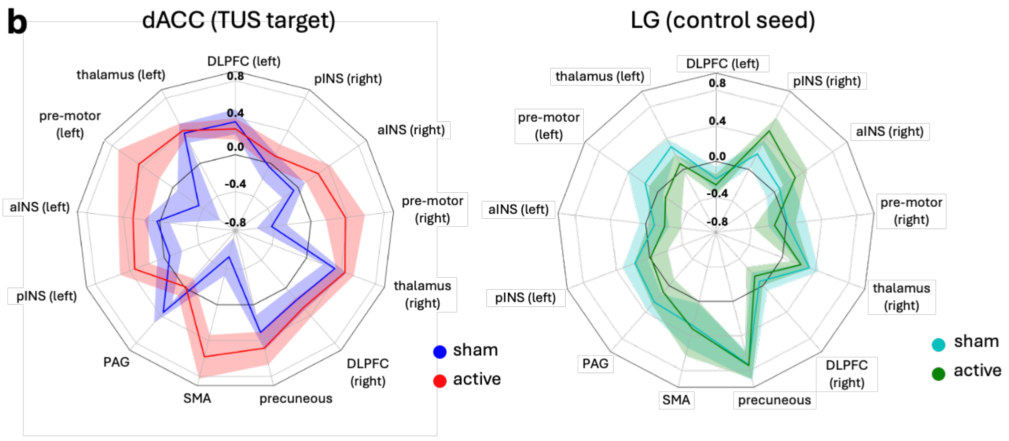

In the updated manuscript, these results are presented in the new Figure 4, with new text added to the results section in *'2.4 Control analyses to assess specificity of TUS effects to the dACC region only'* and new text added into the methods to describe the methodology used, and to the discussion in relation to these additions.

2. Since the reported reduction in pain ratings between 28 and 55 minutes in the active group lacks direct supporting evidence, and the statistical significance ($p = 0.049$) is marginal and would not withstand correction for multiple comparisons, the interpretation of the delayed analgesic effect is arbitrary.

The intention is not to overstate the finding, and we agree that the p-value ($p = 0.049$) is marginal and should be interpreted cautiously. Regarding the lack of direct supporting evidence for a reduction in pain ratings, we acknowledge that the overall main effect of the intervention was not significant. Our interpretation is therefore focused specifically on the significant group × time interaction in the final segment of the experiment, rather than on a global effect. The slope difference between 28 and 55 minutes reflects the only interval in which the trajectories of the two groups diverged, which is why we highlighted it as a potential delayed effect rather than a definitive analgesic outcome.

The planned analysis focused on overall condition, time, and interaction effects. The slope comparison was conducted as exploratory analysis to identify where within the time course differences manifested, not as an independent confirmatory test. It is worth noting that the hypothesis for TUS-induced changes is different between timepoints; specifically that TUS-induced effects would not be expected to occur at T0 using this offline study design. So, this does make statistical analyses of this data more complex, for example the multiple comparisons correction would account for the T0 comparison, even though no change would be expected. We only expect to have seen TUS effects on the pain ratings at T1 and T2.

In the manuscript, we have indicated clearly in the Results section that this is exploratory analysis, and the following has been added to the Discussion section (page 14) to address this: '*Since there was not an overall main effect of condition (active or sham TUS) and the statistical significance for the decrease between T1 and T2 is marginal (p=0.049), the potential delayed analgesic effect discussed here requires replication in further studies.*'

3. While the correlation between changes in GABA and pain ratings appears reasonable, there is no evidence that it is attributable to stimulation, given the absence of group differences in

GABA between active and sham. Instead, differences in GABA levels may reflect participants' state at that time, particularly under cold pain exposure.

While we agree that differences in GABA concentration can reflect participants' state, we believe the relationship between GABA and pain ratings in our data is more nuanced than a simple state effect. When pain intensity ratings are treated purely as a state variable and related to inter individual variability in GABA concentration, the association is weak (non significant), as shown in the plot below. This direction is expected and consistent with prior work showing that experimentally induced pain in healthy participants is associated with reduced GABA concentration relative to rest (Cleve et al., 2015).

Critically, however, the relationship shown in Figure 4D is not a simple state association. That analysis explicitly incorporates the experimental condition, active versus sham TUS, and reveals a substantially stronger correlation. Because sessions were tightly matched and the study used a within subject design, the between condition differences in both GABA concentration, expressed as percent change from sham, and pain ratings, expressed as the change in rating slope between T1 and T2 relative to sham, necessarily reflect TUS related effects on both measures.

Although we did not observe a significant main effect of TUS on GABA concentration or pain ratings at the group level, both measures exhibited substantial inter individual variability. This variability is evident in Figure 4B, which shows a wide range of GABA changes across participants. Within this variability, Figure 4D demonstrates a systematic relationship: participants showing the largest TUS induced decreases in GABA also exhibited the largest modulation of pain ratings, whereas participants with little change or increases in GABA showed correspondingly minimal effects on pain. This pattern is not explained by a generic state effect and instead supports a condition dependent coupling between TUS induced neurochemical changes and behavioural outcomes.

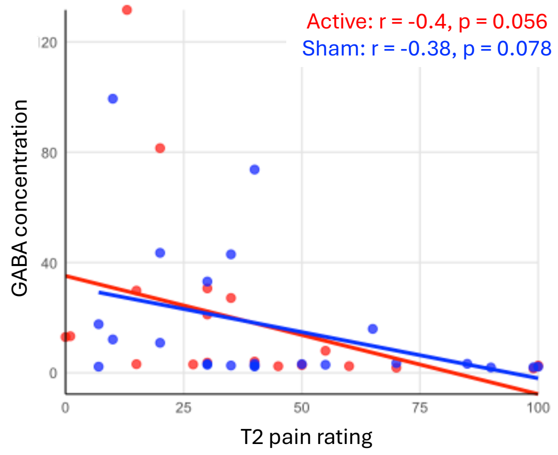

References

Cleve M, Gussew A and Reichenbach J R. (2015). In vivo detection of acute pain-induced changes of GABA+ and Glx in the human brain by using functional 1H MEGA-PRESS MR spectroscopy. Neuroimage. 105;67-75. https://doi.org/10.1016/j.neuroimage.2014.10.042

4. The length of resting-state fMRI (~6 min) is likely insufficient to capture reliable FC changes as suggested by recent work (Ooi et al., 2025). Reference: Ooi, Leon Qi Rong, et al. "Longer scans boost prediction and cut costs in brain-wide association studies." Nature (2025): 1-10.

This is an important point regarding the duration of the resting-state fMRI acquisition. We fully agree that studies, including Ooi et al. (2025), demonstrate that longer resting-state scans generally provide more reliable estimates of brain-wide functional connectivity, particularly in high-dimensional, exploratory analyses such as brain-wide association studies.

The reason that the scan length was constrained to 6.5 minutes in our study was because it was conducted during the tonic cold pain task, and a longer scan duration would have been intolerable for participants due to the discomfort associated with prolonged pain exposure. There could also be a risk of habituation effects with longer duration of cold exposure. This is highlighted by Medina et al. (2025) in a recent study characterising this tonic cold pain task.

Although we acknowledge the short scan duration is a limitation, to ensure that 6.5 minutes would be sufficient we took steps to maximise signal to noise ratio, including use of a short TR (1.5s), multiband and multi-echo approaches, aiming to optimally balance data quality with the experimental constraints associated with the duration of the pain task. Further, recent work shows that spatially constrained or network-informed approaches—including seed-based functional connectivity or network-level analyses—can yield reasonably reliable estimates from short acquisitions. For example, Duda et al. (2023) demonstrated that 5 to 6 minute scans can produce stable network estimates when spatial priors or network constraints are applied. These methods are more closely aligned to the seed-based functional connectivity approach used in the current study. In addition, recent work from our own group, with data collected at the same centre and using the same analysis approaches, has demonstrated functional connectivity changes with a comparable scan duration of 5 minutes (Yaakub et al. 2023). We also emphasise that other elements of the study design are optimised for increasing reliability, including the within-subject design and reasonable sample size.

We have added the following section to the discussion to cover this topic: *"The relatively short scan duration of 6.5 minutes was required to ensure tolerability of the tonic cold stimulus for the length of the scan block; however, this is a limitation, with recent studies demonstrating that longer scans improved reliability in brain-wide association studies[29.]"*

References:

Duda, M., Iraji, A., Ford, J. M., Lim, K. O., Mathalon, D. H., Mueller, B. A., Potkin, S. G., Preda, A., Van Erp, T. G. M., & Calhoun, V. D. (2023). Reliability and clinical utility of spatially constrained estimates of intrinsic functional networks from very short fMRI scans. Human Brain Mapping, 44(6), 2620–2635. https://doi.org/10.1002/hbm.26234

Medina, S., and S. W. Hughes. 2025. A Novel Investigation of an In-Scanner Alternative to the Cold Pressor Test in Healthy Individuals. Human Brain Mapping 46, no. 11: e70291. https://doi.org/10.1002/hbm.70291.

Yaakub, S.N., White, T.A., Roberts, J. et al. Transcranial focused ultrasound-mediated neurochemical and functional connectivity changes in deep cortical regions in humans. Nat Commun 14, 5318 (2023). https://doi.org/10.1038/s41467-023-40998-0

5. It may be helpful for the authors to report whether FC differences between the active and sham groups were observed during the first and third runs of rs-fMRI. The first fMRI run is closer to the stimulation and may have a higher likelihood of capturing such FC changes.

Our hypothesis regarding these temporal dynamics would be that any increases in functional connectivity would be stronger in the latest block (run 3 compared to run 1) as in a few studies now it is shown that TUS effects seem to take a bit longer to develop. However, previous results from our lab have also indicated that TUS effects may be dependent on other factors, one of which could be the brain state (Yaakub et al, 2023).

We have now carried out an identical whole-brain seed based analyses on the two resting state blocks (run 1 at ~18 minus post-TUS and run 3 at ~37 mins post-TUS), and there was no significant difference in functional connectivity with the dACC seed region at either of these time points (whole brain, mixed effects analysis, Z>2.3, p<0.05 comparing active vs sham conditions). The fact that there are changes during the pain stimulus but not during rest may indicate some state-dependency of the TUS effects.

In addition, we have created radar plots for showing the difference between the active and sham conditions across the regions shown in Figure 3C, this time for all fMRI blocks (1-3). These plots show the difference is close to zero in block 1 and block 3. There are deviations from zero, showing differences between active and sham conditions, shown during block 2 (tonic cold pain). The same radar plots are also shown for a control seed region (lingual gyrus; LG), illustrating that the differences between the active and sham condition seen with the dACC seed in the tonic pain condition are specific to TUS and not due to a general session effect.

These new analyses and plots are included in the revised manuscript in the new results section '2.5 State-dependency of TUS effects' and in the new Figure 5 (also shown below for completeness), as well as additional text added to the discussion in relation to these findings.

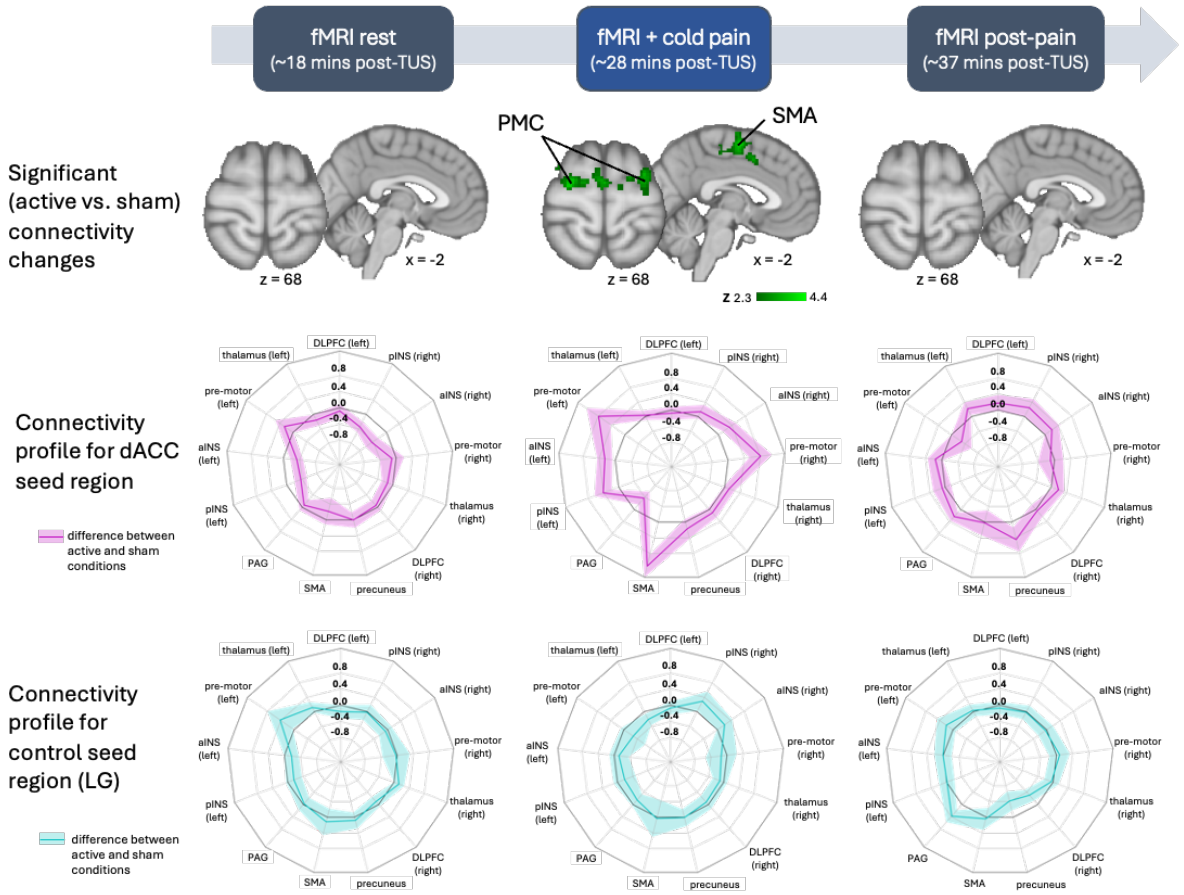

Figure 5: State dependency of TUS effects (N=29)

Significant differences for the active vs. sham comparison in the whole-brain seed-based functional connectivity analysis with the dACC seed for fMRI block 1 (rest; left), fMRI block 2 (tonic cold pain; centre) and fMRI block 3 (post-pain; right) are shown in the top row. There was no significant difference during block 1 or block 3, with altered connectivity between the dACC and SMA and pre-motor cortices during block 2 (tonic cold pain). Radar plots for the difference between active and sham conditions for the dACC connectivity profile are shown in the middle row to illustrate this, with the difference close to zero in block 1 and block 3, and deviations from zero, showing differences between active and sham conditions, shown during block 2 (tonic cold pain). Finally, in the bottom row, the same radar plots are shown for a control seed region (lingual gyrus; LG), illustrating that the differences between the active and sham condition seen with the dACC seed in the tonic pain condition are specific to TUS and not due to a general session effect.

Error bars show the SEM. DLPFC; dorsolateral prefrontal cortex, pINS; posterior insular cortex, aINS; anterior insular cortex, SMA; supplementary motor cortex, PAG: periaqueductal grey.

References:

Yaakub, S.N., White, T.A., Roberts, J. et al. Transcranial focused ultrasound-mediated neurochemical and functional connectivity changes in deep cortical regions in humans. Nat Commun 14, 5318 (2023). https://doi.org/10.1038/s41467-023-40998-0

6. Without an assessment of blinding effectiveness, it is difficult to determine whether participants remained blinded throughout the experiment.

We agree with this and it is acknowledged in the discussion that: '*Assessment of blinding effectiveness for participants would have been valuable to evaluate any bias potentially introduced by participant's expectations.*' Formal assessment of blinding effectiveness will be included in our future study designs.

Although this was not assessed formally, there are two factors we think indicate that our blinding was effective. The first is that we did not tell participants one session was a 'sham' stimulation prior to the completion of the sessions. The information sheet simply outlined that TUS would be administered at the experimental sessions. This was done to ensure that participants were not alerted to the fact one session was 'real' and the other 'fake', to avoid them trying to guess which session was which and therefore biasing any responses. This has been added to the Discussion section: *'For participants, they were not informed prior to completion of the study that there was a 'sham' session, and the sound delivered via bone conducting headphones during the sham session aimed to closely match the active TUS.'* Secondly, but related to the first point, participants received a debrief at the end of the final session, at which they were told that one of the sessions had involved a 'sham' stimulation. Most participants expressed surprise at this stage and stated that they were unaware that we had a sham condition. We therefore believe that the majority of participants were sufficiently blinded to the study conditions. Additionally, studies implementing the same blinding procedure in our lab have assessed blinding effectiveness for participants, with high rates of effectiveness (for example, see Yaakub et al. 2023).

References:

Yaakub, S.N., White, T.A., Roberts, J. et al. Transcranial focused ultrasound-mediated neurochemical and functional connectivity changes in deep cortical regions in humans. Nat Commun 14, 5318 (2023). https://doi.org/10.1038/s41467-023-40998-0

Additional changes to the manuscript (not requested by reviewers)

While making the requested changes we have also made a change to Figure 3C which we felt made the plot clearer. We have included more brain regions in the radar plot, also splitting the regions previously included more accurately (such as including left and right regions separately, rather than the sinlge bilateral region).

REVIEWERS' COMMENTS

Reviewer #1 (Remarks to the Author):

Thank you for your thorough and thoughtful responses. There are a few remaining questions to be addressed:

Introduction
1. The phrasing of the new hypothesis is currently a bit confusing. I would recommend rewriting as "During painful heat stimulation, ACC Glx increases and GABA decreases; therefore, during TUS-induced analgesia (decreased pain intensity), we hypothesized that ACC Glx would decrease and GABA would increase."
This has been amended in the manuscript to the suggested wording.

Methods
1. The authors responded that the multi-focal approach was used to maximize target engagement since TUS is focally specific, which highlights the importance of the specific target site within the dACC. Can you add dACC target site information/coordinates to the new Supplementary Table 3 for the previous dACC TUS studies? Were there meaningful differences in the dACC sites chosen for this study compared to other dACC TUS studies which found analgesic effects?
Information about the stimulation site for each study has been added to Supplementary Table 3. For the ACC studies, one study (Strohman et al) used a target posterior to the targets used in this study. The other used 8 targets across the ACC and initially targeted all 8 to test for immediate symptom reduction, before administering longer stimulations to the 4 targets that produced the strongest reduction. The locations of some of these targets are more similar to those used in this study (located in the more anterior part of the dACC), although this study also included targets in the sgACC, which is not similar.

2. Since the three dACC TUS sites were always stimulated in the same order in each subject, is there any potential for order effects?
There is no potential for order effects because the three sites are not being assessed individually as interventions – if we were assessing the effects of TUS to site A, site B and site C separately then it would be an issue that the order of the sites was not randomised. However, the intervention being assessed was TUS applied to the three sites, which was the same intervention for all participants. This was applied in a counterbalanced design across the two conditions included in the study (active TUS and sham TUS).
We do agree that this particular profile of sonication within the dACC (A then B then C) may have produced the outcome we observed, and a different order may have produced a different outcome, but this cannot be known and is outside the scope of the current study.

3. Thank you for providing more rationale for the sonication parameters. My question remains about the choice to sonicate for 80 seconds.
The study was conducted when ITRUSST was still discussing the parameters for safety limits, with absolute thermal rise limited to 2 degrees in the head (including the skull, soft tissues and brain), and therefore we constrained the sonication length to 80s because in some rare cases this limit was being exceeded. Given that the ITRUSST safety guidelines have now been finalised and that we are well within the thermal dose limit, the sonication length could have been extended.

Reviewer #2 (Remarks to the Author):

I co-reviewed this manuscript with one of the reviewers who provided the listed reports. This is part of the Nature Communications initiative to facilitate training in peer review and to

provide appropriate recognition for Early Career Researchers who co-review manuscripts.

Reviewer #3 (Remarks to the Author):

The authors have successfully addressed all comments.

Reviewer #4 (Remarks to the Author):

I would like to thank the authors for their thorough revision of the manuscript. They have addressed all of my concerns.
